# The E3 ubiquitin ligase MG53 inhibits hepatocellular carcinoma by targeting RAC1 signaling

**DOI:** 10.1038/s41389-022-00414-6

**Published:** 2022-07-20

**Authors:** Xiaomin Ma, Xiaoxiao Ma, Lihui Zhu, Yunxue Zhao, Mengmeng Chen, Tao Li, Yueke Lin, Dapeng Ma, Caiyu Sun, Lihui Han

**Affiliations:** 1 Shandong Provincial Key Laboratory of Infection & Immunology, Shandong Provincial Clinical Research Center for Immune Diseases and Gout, Department of Immunology, School of Basic Medical Sciences, Cheeloo college of Medicine, Shandong University, Jinan, 250012 China; 2 Department of General Surgery, The First Affiliated Hospital of Shandong First Medical University & Shandong Provincial Qianfoshan Hospital, Jinan, 250014 Shandong Province China; 3 Department of Pharmacology, Shandong University School of Basic Medical Sciences, Jinan, 250012 China; 4 Qingdao Restore Biotechnology Co., Ltd, Qingdao, Shandong 266111 P. R. China; 5 Department of Infectious diseases, Shandong Provincial Hospital Affiliated to Shandong First Medical University, Jinan, 250021 China

**Keywords:** Tumour biomarkers, Cell signalling, Ubiquitylation

## Abstract

Ras-related C3 botulinum toxin substrate 1 (RAC1) overexpressiosn and hyperactivation are correlated with aggressive growth and other malignant characteristics in a wide variety of cancers including hepatocellular carcinoma (HCC). However, the regulatory mechanism of RAC1 expression and activation in HCC is not fully understood. Here, we demonstrated that E3 ubiquitin ligase MG53 (also known as tripartite motif 72, TRIM72) acted as a direct inhibitor of RAC1, and it catalyzed the ubiquitination of RAC1 and further inhibited RAC1 activity in HCC cells. Mechanistically, MG53 directly bound with RAC1 through its coiled-coil domain and suppressed RAC1 activity by catalyzing the Lys48 (K48)-linked polyubiquitination of RAC1 at Lys5 residue in HCC cells. We further demonstrated that MG53 significantly suppressed the malignant behaviors of HCC cells and enhanced the chemosensitivity of HCC cells to sorafenib treatment by inhibiting RAC1-MAPK signaling axis. In summary, we identified MG53 as a novel RAC1 inhibitor and tumor suppressor in HCC, and it suppressed HCC progression by inducing K48-linked polyubiquitination of RAC1 and further inhibiting the RAC1-MAPK signaling. Altogether, our investigation provided a new therapeutic strategy for RAC1 overactivated tumors by modulating MG53.

## Introduction

Hepatocellular carcinoma (HCC) is a common malignant tumor and it is one of the leading causes of cancer-related mortality worldwide [1]. Most of the HCC patients are diagnosed at advanced stages and the common therapies for HCC are restricted to surgical interventions, local ablative therapies, and chemotherapy [2]. Sorafenib is the most commonly used first-line chemotherapy drug for the advanced HCC patients. However, the current therapeutic effect on HCC is still unsatisfactory because of the high frequency of resistance to the treatment [3]. Therefore, it is urgent to understand the pathological mechanisms of HCC and provide the novel therapeutic strategies for HCC patients.

RAC1, a well-studied and highly conserved member of the RHO family of small GTPases, acts as a molecular switch in cells, active when bound to GTP and inactive when bound to GDP [4]. RAC1 regulates diverse cellular processes including cell proliferation, metabolism, reactive oxygen species (ROS) production, cytoskeletal reorganization, and inflammatory responses [5–7]. RAC1 has recently emerged as a critical regulator of tumor and a promising therapeutic target for cancer drug discovery. RAC1 is dysregulated in a variety of tumors including HCC, and the overexpression and hyperactivation of RAC1 are correlated with aggressive growth and other malignant characteristics of cancer cells [8–10]. Deletion of RAC1 gene in cancer significantly reduced the tumor formation in the respective tissues, which indicated the critical involvement of RAC1 in tumor progression [9, 10]. RAC1 activation is regulated through a number of mechanisms, including modification at the posttranslational level [11–14]. It is reported that XIAP and cIAP1 directly interacted with RAC1 in a nucleotide-independent manner in vitro and in vivo, and further directly catalyzed ubiquitous degradation of RAC1 [15]. Besides, HACE1 preferentially interacted with GTP-bound RAC1 and catalyzed its polyubiquitylation [16]. RAC1 can also be ubiquitinated by TRAF6 in a murine ischemia-reperfusion model [17]. Though the RAC1 activity was intensively studied [18–20], its regulatory mechanism in HCC remained to be clarified.

In this study, we identified mitsugumin 53 (MG53) as a novel negative regulator of RAC1 in HCC. MG53 (also known as tripartite motif 72, TRIM72) was originally reported to be essential for maintaining the integrity of skeletal muscle plasma membrane [21], whereas with the development of the related research, MG53 has been implicated in the regulation of multiple physiological and pathological processes in various tissues through different mechanisms [22–25]. As an E3 ubiquitin ligase, MG53 is reported to play a regulatory role in the process of cell membrane repair, myogenesis and metabolism [21, 24, 26]. It is recognized recently that multiple E3 ubiquitin ligases play critical roles in the transformation, epithelial–mesenchymal transition (EMT), and metastasis of cancers by inducing ubiquitination of its target proteins [27, 28], whereas whether E3 ligase MG53 plays a role in the progression of HCC remains to be clarified.

In this study, we identified MG53 as a novel RAC1 inhibitor and it suppressed HCC progression via its direct interaction with RAC1 and induced K48-linked poly-ubiquitination of RAC1 at Lys5 residue, which further led to the inhibition of RAC1-MAPK signaling. Thus, our study indicated a novel therapeutic strategy for RAC1 overactivated cancers by modulation of MG53.

## Results

### MG53 directly interacted with RAC1

RAC1 is recognized to be dysregulated in a variety of tumors including HCC, and the overexpression and hyperactivation of RAC1 are involved in aggressive cell proliferation and cell mobility [4]. Therefore, inhibiting the expression and activation of RAC1 is critical for the suppression of cancer progression.

**Fig. 1:**
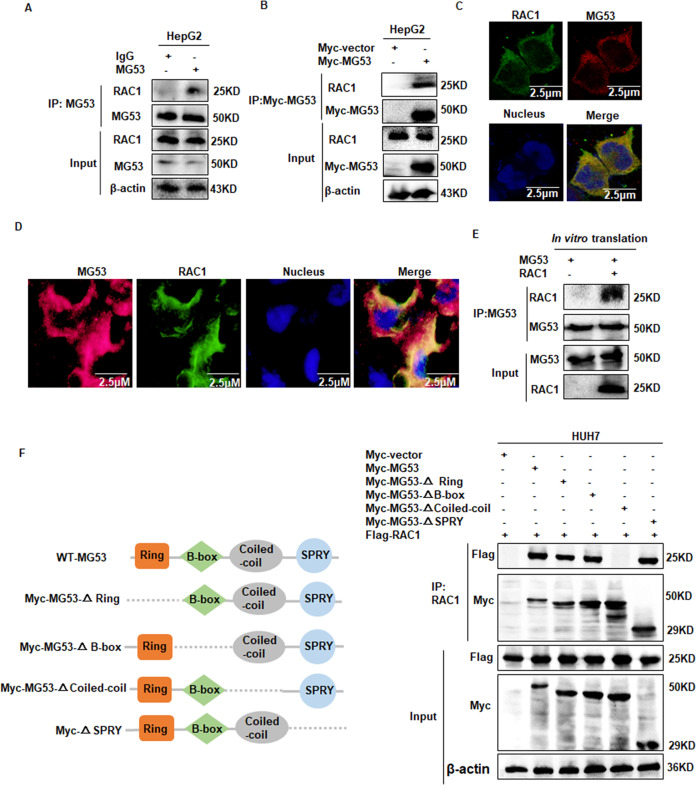
MG53 directly interacted with RAC1 in HCC cells. **A** HepG2 cells were harvested and immunoprecipitation was performed to detect the endogenous interaction between MG53 and RAC1. **B** HepG2 cells were transfected with MG53 plasmid or mock control and further cultured for 24 h. Immunoprecipitation was performed to detect the interaction between Myc-MG53 and RAC1. **C** HepG2 cells were transfected with Myc-MG53 plasmids and further cultured for 24 h. The transfected cells were further fixed and stained with primary antibodies against Myc and RAC1, followed by staining with fluorescence-conjugated secondary antibodies (green for RAC1, red for MG53). DAPI was used to stain the nucleus, and the co-localization between MG53 and RAC1 was visualized as yellow fluorescence in the merged panel. **D** HCC clinical specimen was used to stain with primary antibody against MG53 and RAC1, followed by staining with fluorescence-conjugated secondary antibodies (green for RAC1, and red for MG53). DAPI was used to stain the nucleus, and the co-localization between MG53 and RAC1 was visualized as yellow fluorescence in the merged panel. **E** MG53 protein and RAC1 protein were synthesized by an in vitro translation system. Immunoprecipitation assay was performed to detect the direct interaction between MG53 and RAC1. **F** The truncated mutants of MG53 were constructed, and further co-transfected with Flag-RAC1 plasmid into HUH7 cells. 24 h after the transfection, IP assay was performed to determine the interacting domain of MG53 with RAC1.

MG53 is a member of the E3 ubiquitination ligases and it could induce ubiquitination through direct interaction with its substrates [29]. To investigate the potential role of MG53 in regulating RAC1 stability, we first examined whether MG53 could interact with RAC1 in HCC cells. Co-IP assay showed that MG53 interacted with endogenous RAC1 in HepG2 cells (Fig. 1A, B). Confocal microscopy assay showed that MG53 was co-localized with RAC1 in the cytoplasm of HCC cells (Fig. 1C) and clinical HCC tissues (Fig. 1D). We further demonstrated that MG53 directly interacted with RAC1 in an in vitro protein transcription and translation system (Fig. 1E). To further verify the molecular basis of the direct interaction between MG53 and RAC1, we constructed a series of domain deleted mutants of MG53 for further investigation (Fig. 1F). Co-IP assay showed that the coiled-coil domain deleted mutant of MG53 failed to interact with RAC1, which indicated that MG53 interacted with RAC1 through its coiled-coil domain (Fig. 1F). Altogether, these data demonstrated that MG53 directly interacted with RAC1 in HCC cells via its coiled-coil domain.

### MG53 negatively regulated the expression of RAC1 in HCC cells and clinical HCC specimens

**Fig. 2:**
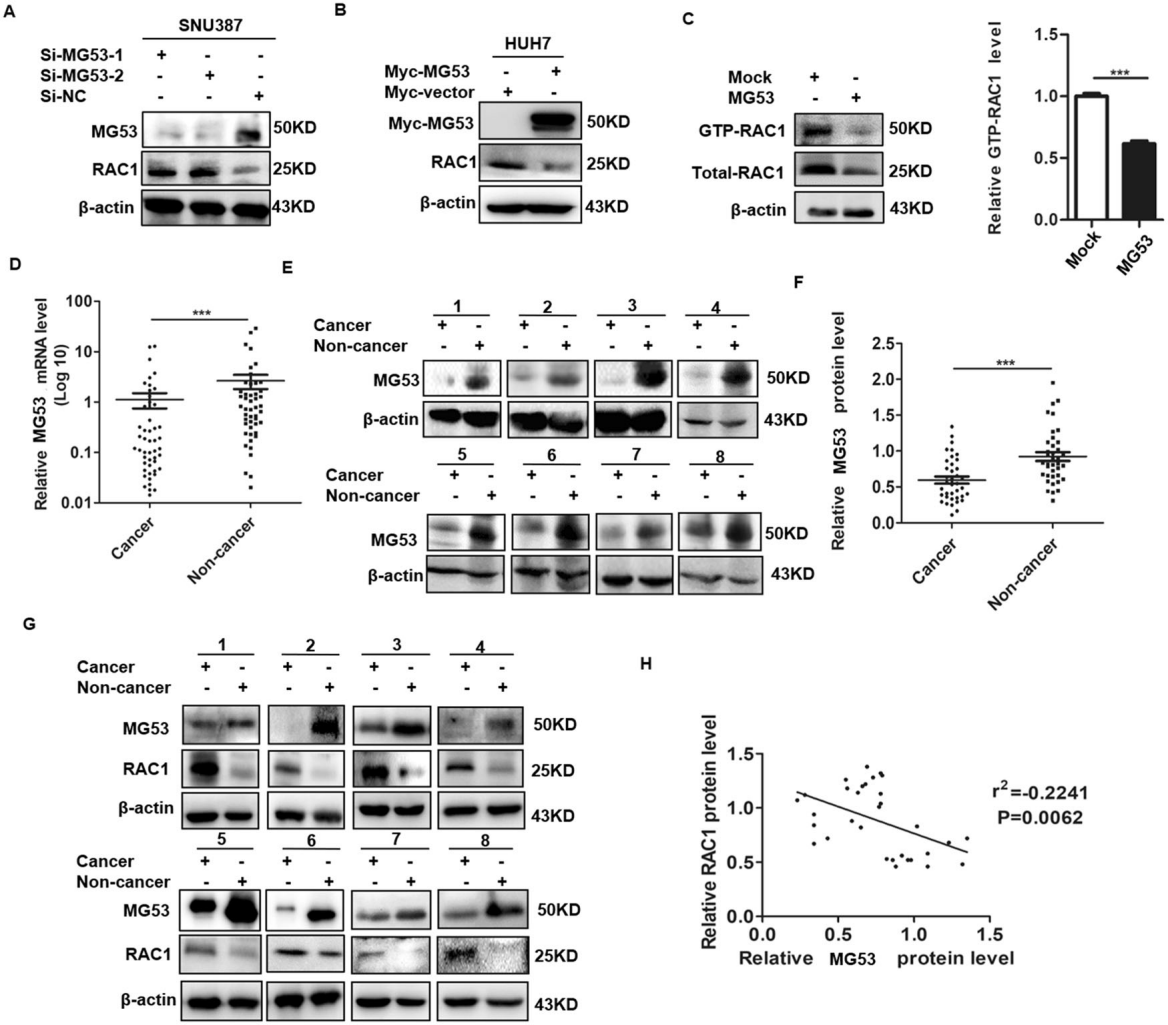
RAC1 expression was downregulated by MG53 in HCC cells and clinical HCC specimens. **A** SNU387 cells were transfected with Si-MG53 or Si-NC and further cultured for 48 h before western blot assay was performed to detect the expression level of RAC1. **B** HUH7 cells were transfected with Myc-MG53, and Myc-vector transfected cells acted as a mock control. 24 h after the transfection, western blot assay was performed to detect the expression level of RAC1. **C** HUH7 cells were transfected with Myc-MG53 or mock control. 24 h after the transfection, the relative levels of GTP-RAC1 and total RAC1 were detected by RAC1 GTPase activity kit. The expression of GTP-RAC1 after overexpression of MG53 were normalized with total RAC1. **D** Expression levels of MG53 mRNA in 51 pairs of HCC tissues and matched non-cancerous liver tissues were detected and statistically analyzed. **E**, **F** Western blot was performed to detect the relative protein levels of MG53 in paired HCC specimens and representative bands were presented (**E**). The relative band densities from all of the detected patients were analyzed by Image J software and normalized by β-actin (**F**). **G**, **H** Expression of MG53 and RAC1 in human hepatocellular carcinoma tissues from 30 HCC patients were detected by western blot (**G**). Correlation between the expression of MG53 and RAC1 was further analyzed (**H**). *P < 0.05, **P < 0.01 and ***P < 0.001 for statistical analysis of the indicated groups.

We have verified the direct interaction between MG53 and RAC1, thus we are further interested in defining whether MG53 have any effect on the expression and stability of RAC1 in HCC cells. Further investigation showed that the level of RAC1 protein was significantly increased after knockdown of MG53 by its specific small interference RNA (Si-MG3), and RAC1 protein was significantly inhibited in the MG53 overexpressed HCC cells (Fig. 2A, B). Our data further showed the mRNA level of RAC1 in HCC cells was not significantly altered after knockdown or overexpression of MG53 (Supplementary Fig. 1), which indicated that RAC1 was negatively regulated by MG53 at the protein level. Like other GTPases, RAC1 switches between an active, GTP-bound state and an inactive, GDP-bound state, and the active form GTP-RAC1 is involved in the regulation of malignant behaviors of tumor cells [18]. To investigate whether MG53 had any effect on the RAC1 activity, RAC1 GTPase activity assay was performed and our data showed that MG53 significantly inhibited RAC1 activity in HCC cells (Fig. 2C). These data indicated that MG53 could significantly downregulate the RAC1 expression and inhibit RAC1 activity in HCC cells.

In consideration of the essential regulatory role of RAC1 in HCC, we further investigated the expression and correlation of MG53 and RAC1 in clinical HCC specimens. We detected MG53 expression by qRT-PCR and western blot in a cohort of clinical specimens including 51 pairs of HCC tissues and corresponding distal non-cancerous liver tissues. The clinicopathologic features of all of these recruited HCC patients were presented in Supplementary Table 1. qRT-PCR assay indicated that MG53 expression was significantly decreased in HCC tissues compared with the matched non-cancerous liver tissues (Fig. 2D). In consistence with qRT-PCR data, western blot assay also showed that MG53 expression was either completely lost or significantly decreased in HCC tissues (Fig. 2E, F). Besides, western blot assay of the expression of RAC1 and MG53 in the matched HCC specimens further verified that the expression levels of MG53 was significantly negatively correlated with those of RAC1 (Fig. 2G, H), which verified the negative regulation of RAC1 by MG53 in clinical HCC specimens. Altogether, these data collectively indicated that MG53-mediated downregulation of RAC1 activity was involved in the HCC progression.

### MG53 catalyzed K48-linked polyubiquitination of RAC1 at Lys5 and further induced RAC1 degradation

**Fig. 3:**
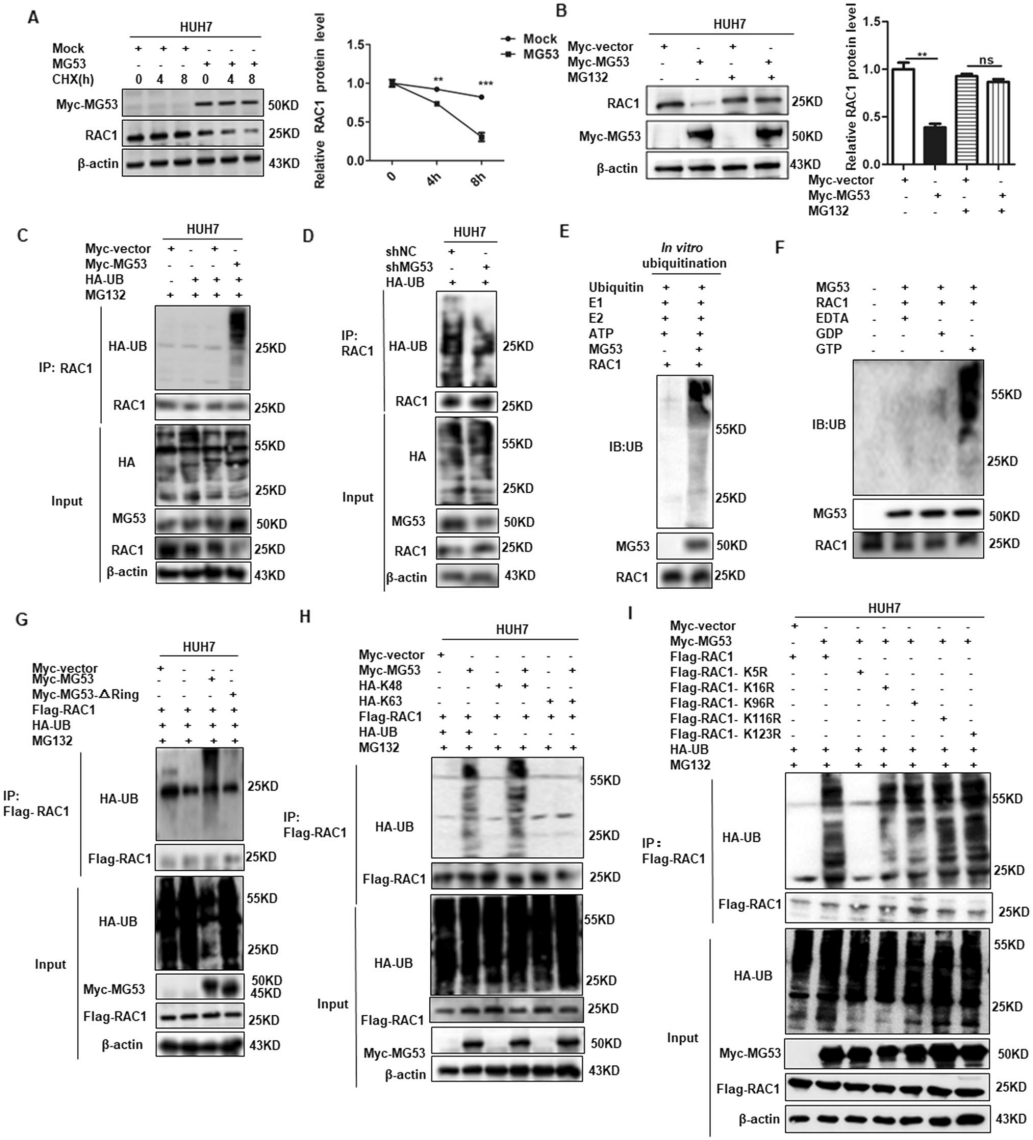
MG53 catalyzed K48-linked polyubiquitination of RAC1 at Lys5 residue in HCC cells. **A** HUH7 cells were transfected with Myc-MG53 plasmid or its mock control. The transfected cells were cultured for 24 h before being further incubated with cycloheximide (CHX) (10 μg/ml) for the indicated time points. The levels of RAC1 at different time points were detected by western blot. Band intensities were quantitatively analyzed and the values were normalized to β-actin. **B** HUH7 cells were transfected with Myc-MG53 plasmid or its mock control. The transfected cells were cultured for 24 h followed with treatment with MG132 (100 nM) for 6 h. Western blot was performed to detect the protein level of RAC1. The relative RAC1 protein level was statistically analyzed from three independent experiments (right panel). **C** HUH7 cells were co-transfected with Flag-MG53 and HA-UB plasmid, and protein was denatured before the ubiquitination assay of RAC1. **D** HUH7 cells were co-transfected with sh-MG53 (or sh-NC) and HA-UB plasmid, and the protein samples were harvested and denatured for subsequent IP assay of ubiquitination. **E** The MG53 and RAC1 proteins obtained by in vitro protein translation assay were mixed with E1, E2 and UB for in vitro ubiquitination assay. **F** Recombinant RAC1 loaded with GTP, GDP, or the Mg^2+^ -free form (EDTA) were used in the ubiquitination reaction, and the ubiquitination of different forms of RAC1 by MG53 were further analyzed. **G** HUH7 cells were transfected with Flag-RAC1plasmid, together with Myc-MG53 plasmid or its RING domain deleted mutant plasmids. The transfected cells were treated with MG132 (100 nM) and the ubiquitination of RAC1 was performed by immunoprecipitation assay after the protein samples were denatured. **H** HUH7 cells were transfected with Flag-MG53 plasmid, together with HA-UB-K48 or HA-UB-K63 plasmid. The transfected cells were treated with MG132 (100 nM) and the protein lysates were harvested and denatured for ubiquitination assay of RAC1. **I** HUH7 cells were co-transfected with Myc-MG53 and Flag-RAC1 mutants (Flag-RAC1-K5R, Flag-RAC1-K16R, Flag-RAC1-K96R, Flag-RAC1-K116R and Flag-RAC1-K116R), and the transfected cells were treated with MG132 (100 nM). The ubiquitination of RAC1 and its mutants were further analyzed after all of the protein samples were denatured. *P < 0.05, **P < 0.01 and ***P < 0.001 for statistical analysis of the indicated groups.

To detect whether MG53 regulated the stability of endogenous RAC1, we measured the half-life of RAC1 following inhibition of de-novo protein synthesis by cycloheximide (CHX). Compared with the mock group, the half-life of RAC1 was significantly decreased in MG53 transfected group (Fig. 3A). The down-regulation of RAC1 by MG53 in HCC cells was effectively abrogated after the treatment with the proteasome inhibitor MG132, which indicated that MG53 negatively regulated RAC1 by proteasome-mediated degradation (Fig. 3B). In consideration of the E3 ubiquitin ligase function of MG53, we are further interested in defining whether MG53 exerts its effect through catalyzing the ubiquitination modification of RAC1. The data showed that MG53 could conjugate the poly-ubiquitination chain to RAC1 (Fig. 3C) and the endogenous ubiquitination assay showed that knockdown of MG53 could abrogate the ubiquitination modification of RAC1 in HCC cells (Fig. 3D), which indicated that MG53 could catalyze RAC1 ubiquitination modification in HCC cells. We further performed the in vitro ubiquitination assay and verified that MG53 induced direct ubiquitous modification of RAC1 in vitro (Fig. 3E), and further investigation showed that MG53 could catalyze the ubiquitination of GTP-RAC1 (Fig. 3F). Altogether, these data indicated that MG53 could negatively regulated RAC1 in HCC cells by directly catalyzing the ubiquitination of GTP- RAC1.

MG53 belongs to the RING-type E3 ligase, and thus we further try to define whether the ubiquitination activity of MG53 was dependent on its RING domain. Our data showed that the RING-domain deleted MG53 mutant could almost completely abrogated the ubiquitination activity (Fig. 3G), which indicated that MG53 induced the poly-ubiquitination modification of RAC1 through its RING domain. Further investigation showed that MG53 promoted the K48-linked poly-ubiquitination of RAC1 but not the K63-linked poly-ubiquitination (Fig. 3H), which indicated that MG53 catalyzed the K48-linked poly-ubiquitination and degradation of RAC1. To further identify the lysine residues responsible for MG53-mediated poly-ubiquitination, we constructed a series of site-directed mutants including RAC1 (K5R), RAC1 (K16R), RAC1 (K96R), RAC1 (K116R) and RAC1 (K123R), in which the lysine residues at positions 5, 16, 96, 116, and 123 were replaced with arginine. Co-IP assay showed that RAC1 (K5R) mutant could significantly abrogate MG53-induced poly-ubiquitination (Fig. 3I), which indicated that MG53 catalyzed the poly-ubiquitination modification of RAC1 at Lys5 residue. Altogether, these data demonstrated that MG53 catalyzed the K48-linked poly-ubiquitination and degradation of RAC1 at Lys5 in HCC cells.

### MG53 exerted the anti-tumor effect on HCC cells

**Fig. 4:**
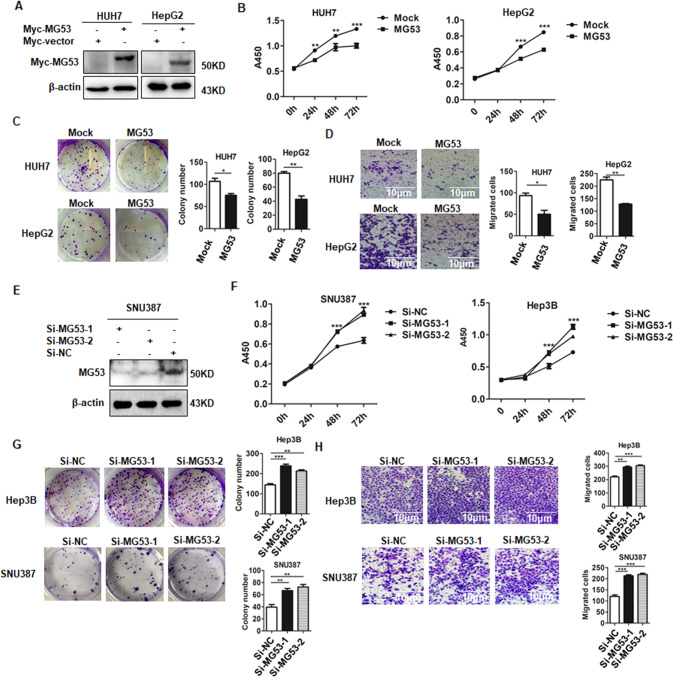
MG53 significantly inhibited the malignant behaviors of HCC cells. **A**–**D** HepG2 cells and HUH7 cells were transfected with Myc-MG53 plasmid, and the Myc-vector plasmid transfected cells acted as the mock control. Western blot assay was performed to detect the successful overexpression of MG53 in HCC cells (**A**). CCK-8 assay was performed to detect the cell proliferation status of these MG53-transfected HCC cells at 0 h, 24 h, 48 h, and 72 h (**B**). Colony formation assay was performed to detect the proliferation of these MG53-transfected HCC cells (**C**). To ensure the stable effect of SiRNA and guarantee the high interference effect, we supplemented 20 nM of siRNA every three days until day 10 during the cloning-forming assay. Transwell assay was performed to detect the migration of these MG53-transfected HCC cells (**D**). **E**–**H** HCC cells were transfected with two SiRNA sequences against MG53 (Si-MG53), and western blot assay was performed 48 h after the transfection to detect the effective knockdown of MG53 (**E**). CCK-8 assay was performed to detect the proliferation of these Si-MG53 transfected HCC cells at 0 h, 24 h, 48 h and 72 h (**F**). Colony formation assay was performed to detect the proliferation of these MG53-transfected HCC cells (**G**). Transwell assay was performed to detect the migration of these Si-MG53 transfected HCC cells (**H**). *P < 0.05, **P < 0.01 and ***P < 0.001 for statistical analysis of the indicated groups.

Previous studies have demonstrated that RAC1 plays an important role in the regulation of tumor progression [9, 30, 31]. As we have identified the suppression of RAC1 by MG53, we hypothesized that MG53 might regulate the malignant behaviors of HCC cells. Thus, we constructed the gain-of-function and loss-of-function models of MG53 to further define the role of MG53 in HCC. MG53 plasmid was transfected into HCC cells to construct the gain-of-function model, and the successful overexpression of MG53 was verified by western blot (Fig. 4A). Further investigation showed that overexpression of MG53 significantly decreased the capabilities of proliferation (Fig. 4B), colony formation (Fig. 4C) and migration (Fig. 4D) of HCC cells. Moreover, Si-RNA against MG53 was transfected to HCC cells to construct the loss-of-function model, and the efficient knockdown of MG53 by two SiRNA sequences was verified by western blot (Fig. 4E). In consistence with the gain-of-function data, after knockdown of MG53, the proliferation, colony formation and migration capabilities of HCC cells were significantly increased (Fig. 4F–H). Altogether, these data collectively demonstrated that MG53 acted as a tumor suppressor and inhibited the malignant behaviors of HCC cells.

### MG53 inhibited the malignant behaviors of HCC via its RING domain-mediated ubiquitination of RAC1 at Lys5 residue

We have demonstrated that MG53 induced poly-ubiquitination modification of RAC1 through its RING domain, and we further try to determine whether MG53 exerts the anti-tumor effect by its RING domain. Our data showed that the RING domain deleted mutant of MG53 abrogated its capabilities of inhibiting proliferation, colony formation and migration of HCC cells (Fig. S2 A-C). We have verified that MG53 catalyzed the K48-linked poly-ubiquitination and degradation of RAC1 at Lys5, and we further tried to define whether MG53 exerted its anti-tumor effect via its ubiquitous modification at Lys5 residue of RAC1. We co-transfected HCC cells with MG53 and the series of RAC1 mutants (K5R, K16R, K96R, K116R, and K123R), then detected the malignant behaviors of HCC cells. Our data showed that RAC1-K5R failed to rescue the MG53-mediated inhibiting effect on cell proliferation, colony formation and migration (Fig. S2 D–F), which verified that MG53 exerted its anti-tumor effect via its modification of RAC1 at Lys5 residue. All these data demonstrated that MG53 inhibited the malignant behaviors of HCC cells via its RING domain and abrogated RAC1 activity at Lys5 residue.

### MG53 suppressed HCC via inhibiting RAC1-MAPK signaling axis

**Fig. 5:**
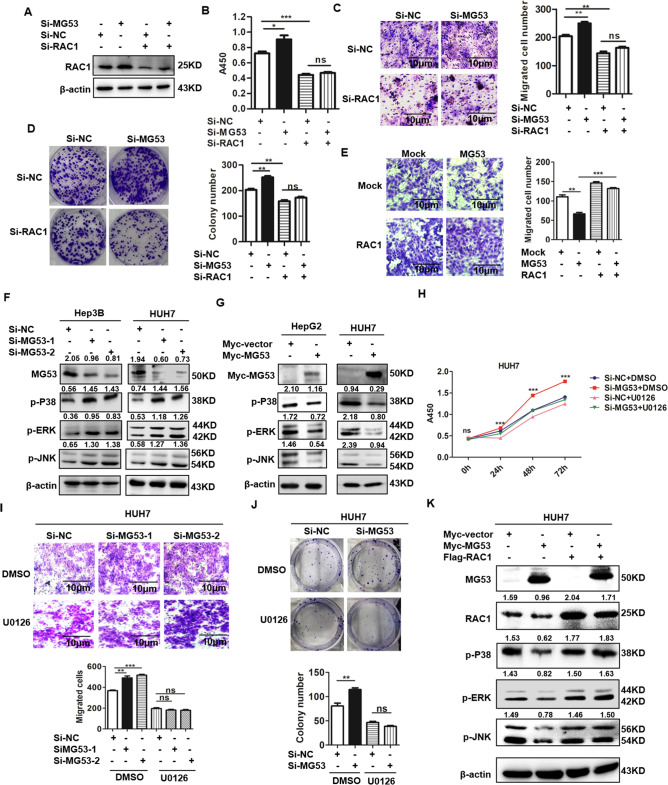
MG53 exerted its anti-tumor effect by inhibiting RAC1-MAPK axis in HCC. **A**–**D** SNU387 cells were co-transfected with Si-MG53 and Si-RAC1. 48 h after the transfection, cells were collected for the detection of RAC1 by western blot (**A**). CCK-8 assay was performed to detect the cell proliferation of these transfected cells at 48 h (**B**). Transwell assay was performed to detect the invasion of these transfected HCC cells (**C**). Colony formation assay was performed to detect the proliferation of these transfected HCC cells (**D**). **E** HUH7 cells were co-transfected with Myc-MG53 plasmid and Flag-RAC1 plasmid. Transwell assay was performed to detect the invasion of these transfected HCC cells. **F** HCC cells were transfected with two SiRNA sequences against MG53 or its mock control. 48 h after the transfection, western blot was performed to detect the levels of p-P38, p-JNK and p-ERK. **G** HCC cells were transfected with Myc-MG53 plasmid or its mock control, and western blot assay was performed to detect the levels of p-P38, p-JNK and p-ERK. **H–J** HUH7 cells were transfected with Si-MG53 to knockdown the expression of MG53, and U0126 (1 μM) was added to inhibit the activation of MAPK signaling pathway. CCK-8 assay was performed to detect the proliferation of these HCC cells at 0 h, 24 h, 48 h and 72 h (**H**). Transwell assay was performed to detect the invasion of these transfected HCC cells (**I**). Colony formation assay was performed to detect the proliferation of these transfected HCC cells (**J**). **K** HUH7 cells were co-transfected with Myc-MG53 plasmid and Flag-RAC1 plasmid and further cultured for 24 h before western blot was performed to detect the expression level of p-P38, p-JNK and p-ERK. *P < 0.05, **P < 0.01 and ***P < 0.001 for statistical analysis of the indicated groups. *P < 0.05, **P < 0.01 and ***P < 0.001 for statistical analysis of the indicated groups.

We have identified a tumor suppressor role of MG53 and its negative regulation of RAC1 signaling, and then we further try to demonstrate whether the anti-tumor effect of MG53 is mediated through RAC1-MAPK signaling. Thus, we transfected siRNA against RAC1 into the MG53-inhibited HCC cells and further detected the malignant behaviors of these transfected cells. Our data showed that after inhibiting the RAC1 levels in HCC cells (Fig. 5A), the aggressive malignant behaviors induced by loss of MG53 were dramatically suppressed (Fig. 5B–D). Besides, after reconstitution of RAC1 in MG53-overexpressed HCC cells, the anti-tumor effect of MG53 was significantly rescued (Fig. 5E), which further demonstrated that MG53 exerted its anti-tumor effect by inhibiting RAC1 in HCC cells.

RAC1 usually exerted its effect through activating its downstream signaling, thus we tested several possibly involved signaling and identified the mitogen-activated protein kinase (MAPK) signaling pathway as the downstream effector signaling of RAC1 in HCC cells. Our data showed that knockdown of MG53 significantly increased the activation of MAPK signaling pathway (Fig. 5F), while overexpression of MG53 dramatically inhibited MAPK pathway activation (Fig. 5G). Besides, when we blocked MAPK pathway by its specific inhibitor U0126, the promotive effect of Si-MG53 on the malignant behaviors of HCC cells was also significantly reversed (Fig. 5H–J), which indicated that MG53 exerted the anti-tumor effect through its effective inhibition of MAPK pathway. Furthermore, when we transfected the HCC cells with both RAC1 and MG53 plasmids, the inhibition of MAPK pathway by MG53 was also significantly reversed (Fig. 5K), which indicated that MG53-induced MAPK pathway inhibition was at the downstream of its negative regulation of RAC1. Altogether, these data collectively demonstrated that MG53 exerted its anti-tumor effect on HCC cells through its effective inhibition of RAC1-MAPK signaling axis.

### MG53 significantly enhanced the chemosensitivity of HCC cells to sorafenib treatment

Sorafenib, an oral multi-kinase inhibitor, remains the most common FDA-approved systemic therapeutic strategy for patients with advanced HCC [3]. Although sorafenib treatment has led to a significant prolonged overall survival time of a huge amount of advanced HCC patients, the high relapse rate and secondary drug resistance remained as the insurmountable obstacles for its clinical application [32, 33]. The aberrant activation of MAPK pathway was reported to be a major cause of sorafenib resistance in HCC patients [34, 35]. Since we have demonstrated that MG53 exerted the anti-tumor effect by inhibiting RAC1-MAPK pathway, thus we further try to clarify whether MG53 could play a role in the response to sorafenib treatment in HCC.

Drug sensitivity assay showed that MG53 significantly increased the response of HCC cells to sorafenib treatment (Fig. S3A). Besides, the treatment of HCC cells by the combination of MG53 and sorafenib significantly inhibited the proliferation (Fig. S3B) and colony formation capabilities (Fig. S3C) of HCC cells. Western blot assay verified that MG53 promoted the response of HCC cells to sorafenib treatment by inhibiting RAC1-MAPK pathway (Fig. S3D). Further investigation showed that MG53 enhanced the chemosensitivity to sorafenib by its RING domain in HCC cells (Fig. S3E, F). Altogether, these data demonstrated that MG53 significantly enhanced the chemosensitivity of HCC cells to sorafenib treatment via inhibiting RAC1-MAPK signaling.

### MG53 significantly inhibited the tumorigenesis of HCC in vivo

**Fig. 6:**
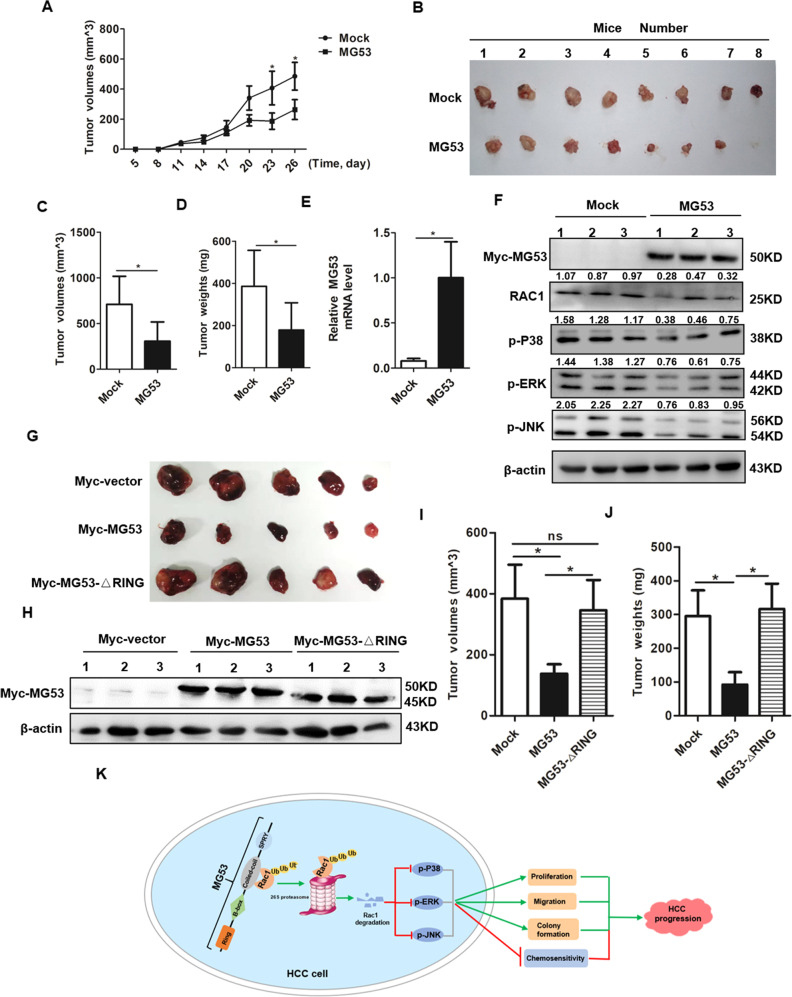
Exogenous overexpression of MG53 inhibited the hepatocarcinogenesis in vivo. To construct xenograft tumor model, 0.1 ml of 10^7^ HUH7 cells were subcutaneously injected to both flanks of the nude mice (4-week-old male BALB/c nude mice, *n* = 8). When visible tumors appeared, the tumors at the left flanks were injected with Myc-MG53 plasmid while tumors at the right flanks were injected with Myc-vector plasmid. The mice were sacrificed on day 26 after the first injection, and the tumors were separated from both flanks of the mice for further analysis. **A**, **B** The tumor growth curve (**A**) and the image of the isolated tumors from sacrificed mice (**B**) were presented. **C**, **D** The tumor volumes (**C**) and tumor weights (**D**) of the indicated groups were analyzed and compared. **E** qRT-PCR was performed to detect the relative MG53 expression level in MG53 and mock plasmid transfected group. **F** Protein extracted from the tumors was used for western blot assay to detect the expression levels of Myc, RAC1, p-JNK, p-P38 and p-ERK. **G**–**J** Another group of xenograft tumor model was constructed as the above described (4-week-old male BALB/c nude mice, *n* = 5 for indicated group). When visible tumors appeared, the tumors were divided into three groups including Myc-vector transfected group, Myc-MG53 transfected group and Myc-MG53-ΔRING transfected group. The tumors were injected with 20 μg of indicated plasmids every other day. The mice were sacrificed on day 28 after the first injection, and the tumors were separated from the nude mice for further analysis. The image of the isolated tumors from sacrificed mice was presented (**G**). **H**–**J** Western blot was performed to detect the successful transfection of the indicated groups (**H**). Tumor volumes (**I**) and tumor weights (**J**) of the indicated groups were analyzed and compared. **K** The working model showing the role and mechanism of MG53 in HCC cells. MG53 directly binds with RAC1 through its coiled-coil domain and further induces ubiquitous degradation of RAC1 by K48 linked poly-ubiquitination, which leads to inhibition of the RAC1-MAPK pathway; and finally reverses the malignant behaviors of HCC cells and enhances the chemosensitivity of HCC cells to sorafenib. *P < 0.05, **P < 0.01 and ***P < 0.001 for statistical analysis of the indicated groups.

To further verify the anti-tumor effect of MG53, we constructed the xenograft HCC tumor model as previously described [36]. When visible tumors appeared, tumors at the left flanks were injected with 20 μg of MG53 plasmid while tumors at the right flanks were injected with 20 μg of vector plasmid every other day until the mice were sacrificed on day 26 after the first injection. Tumor growth curve indicated that MG53 transfected group had a significantly slower growth rate compared with the mock group (Fig. 6A). The tumor sizes, volumes and weights of the MG53 transfected group were all significantly decreased compared with those of the mock group (Fig. 6B–D). qRT-PCR assay verified the successful overexpression of MG53 in the MG53 transfected tumors (Fig. 6E). Western blot assay showed that MG53 transfected group had much lower levels of RAC1, p-JNK, p-ERK and p-P38 (Fig. 6F), which was in consistence with our in vitro data and it further verified that MG53 inhibited the tumorigenesis of HCC through its effective inhibition of RAC1-MAPK signaling pathway. Besides, we also constructed xenografted tumor model by MG53 mutant (RING domain deletion) transfected HCC cells to further validate whether MG53 exerted the tumor suppression function via its RING domain. The tumors isolated from the nude mice were presented and further analyzed (Fig. 6G). Western blot was performed to verify the successful overexpression of MG53 or its mutant in these transfected tumors (Fig. 6H). Our data showed that the MG53 mutant (RING domain deletion) transfected tumors have larger tumor volumes and heavier tumor weights compared with the MG53 transfected tumors, while these MG53 mutant transfected tumors have no significant difference compared with the mock group (Fig. 6I, J). These in vivo data further validated that MG53 acted as a tumor suppressor through its RING domain.

Altogether, in this study we demonstrated that MG53 exerted its anti-tumor effect by ubiquitination mediated degradation of RAC1 atLys5 residue and further inhibiting the RAC1-MAPK signaling pathway in HCC, which finally reversed the malignant behaviors of HCC cells and enhanced the chemosensitivity of HCC cells to sorafenib treatment (Fig. 6K).

## Discussion

RAC1, as an important member of the Rho-GTPase family, is a pleiotropic regulator of many cellular processes, including the cell cycle progression, cell-cell adhesion, motility, and epithelia differentiation [4]. The regulation of RAC1 is important for the manipulation of cancer and has attracted much attention in recent years. Though RAC1 activity is known to be primarily regulated by GEF, GAPs, and GDIs, recent studies showed that post-translational modifications of RAC1 including carboxymethylation, palmitoylation and ubiquitination, also contributed to the regulation of RAC1 activity and function [12, 37, 38]. In this study, we identified MG53 as a novel regulator of RAC1 and verified that MG53 exerted its anti-tumor effect by directly interacting with RAC1 and inducing the ubiquitination-mediated degradation of RAC1 in HCC.

MG53 is highly conserved among different species and it was originally viewed as an essential component of the cell membrane repair machinery [21]. However, MG53 is also a member of the tripartite motif (TRIM) family and it possesses a typical RING domain with effective E3 ligase activity [26]. Multiple E3 ubiquitin ligases are reported to be involved in the regulation of pathogenesis in cancer [39, 40], whereas whether MG53 plays a role in HCC is not known and has been investigated in this study for the first time. Here, we reported that MG53 directly interacted with RAC1 through its coiled-coil domain, and further catalyzed the K48-linked polyubiquitination of RAC1 at Lys5 residue via its RING domain, leading to the proteasomal degradation of RAC1 in HCC cells. The clinical investigation data showed that both of the MG53 mRNA and protein levels were significantly downregulated in HCC tissues compared with non-cancerous liver tissues, and the levels of MG53 and RAC1 was significantly negatively correlated, which indicated that the negative regulation of RAC1 might be involved in the progression of hepatocarcinogenesis.

RAC1 activity is recognized to play a pivotal role in cancer including HCC, thus it is plausible that MG53-induced negative regulation of RAC1 might be critically involved in the progression of HCC. Therefore, we further investigated the effect of MG53-mediated ubiquitination of RAC1 in the malignant behavior of HCC. As expected, both the loss-of-function and gain-of-function cellular model showed that MG53 significantly inhibited the capabilities of proliferation, colony formation and migration of HCC cells, and further investigation demonstrated that the anti-tumor effect of MG53 was mediated through its negative regulation of RAC1 activity. Besides, our further investigation demonstrated that MG53 inhibited the malignant behaviors of HCC via its RING domain-mediated ubiquitination of RAC1 at Lys5 residue. The tumor suppressor role of MG53 was further investigated in the xenograft tumor model, which verified its significant anti-tumor effect on HCC. Then the downregulation of MG53 in HCC cells was verified in the clinical HCC specimens, and the molecular mechanism of its dysregulation in HCC specimens will be further investigated in our future study. Altogether, these data collectively indicated that dysregulation of MG53-mediated abnormal RAC1 expression was involved in the progression of HCC.

The RAC1 protein has been recognized as a central signaling hub that is required for many oncogenes induced transformation, and RAC1 propagates its downstream biological effects by activating its effector signaling pathways [7, 41]. Though RAC1 was reported to be regulated by multiple proteins, the negative regulation of RAC1 activity by MG53 in HCC was reported here for the first time. Here we demonstrated that MG53 induced poly-ubiquitous degradation of RAC1 and further inhibited RAC1 and its downstream mitogen-activated protein kinase (MAPK) pathway. MAPK signaling is composed of a series of evolutionarily conserved kinase modules that link extracellular signals to the machinery controlling fundamental cellular processes including growth, proliferation, differentiation, migration and apoptosis [42]. Though RAC1-MAPK axis has been demonstrated to be involved in the progression of several types of cancers [43–45], the efficient inhibition of RAC1-MAPK axis by MG53 was reported here for the first time.

Chemotherapy is one of the most common treatments for HCC patients, especially for advanced HCC [2]. Despite the emergence of multiple target drugs for HCC in recent years, sorafenib, an orally available tyrosine kinase inhibitor (TKI), still remains the most effective systemic therapy for advanced HCC patients [33]. However, primary and secondary resistance to sorafenib treatment remains the obstacle for the efficacy of sorafenib in HCC [32]. Given that activation of MAPK signaling contributes to the resistance to multiple chemotherapies, especially is responsible for the resistance to sorafenib treatment for HCC, thus we further investigated whether blocking MAPK signaling by MG53-mediated RAC1 inhibition could reverse the resistance of HCC cells to sorafenib treatment. As expected, our data demonstrated for the first time that MG53 significantly enhanced the chemosensitivity of HCC cells to sorafenib by inhibiting the RAC1-MAPK signal axis. Thus, this study indicated a great potential for clinical application of MG53 for enhancing the treatment efficiency of chemotherapeutic drugs in cancer.

In conclusion, in this study we defined E3 ubiquitous ligase MG53 as a novel inhibitor of RAC1 for the first time. We demonstrated that MG53 inhibited HCC progression and enhanced chemosensitivity through directly targeting RAC1 for ubiquitination modification at Lys5 residue, which further led to the suppression of RAC1 activity and inhibition of the downstream MAPK pathway. Thus, this study facilitated further elucidation of the molecular mechanism involved in the malignant progression of HCC and provided a novel therapeutic strategy for RAC1 overactivated tumors.

## Materials and methods

### Cell lines and reagents

The hepatocellular carcinoma cell lines including HepG2 cells, Hep3B cells, SNU387 cells and HUH7 cells were obtained from the Cell Bank of Chinese Academy of Science (Shanghai, China). The culture conditions of the HCC cells were as previously described [46]. Cycloheximide (CHX) was purchased from ApexBio technology company (Houston, Texas, USA). ERK inhibitor (U0126) and sorafenib were purchased from Selleck Chemicals (Houston, Texas, USA). All the cell lines were cytogenetically tested and authenticated by STR profiling within 2 years.

### Plasmids, small interference RNA (SiRNA) and transfection

HCC cells were transfected with plasmid or small interference RNA (SiRNA) according to the previously described procedure [47]. The pCMV-Myc-MG53 plasmid and pCMV-Flag-RAC1 plasmid were purchased from Vigene Biology (Jinan, Shandong, China). SiRNA sequences against MG53 and RAC1 were synthesized by Sigma-Aldrich (Merk, Darmstadt, Germany).

### Co-immunoprecipitation (Co-IP) and western blot

Co-IP was performed and evaluated as previously described [48]. Briefly, cold PBS was used to wash the cells three times, and then the cells were scraped into lysis buffer containing complete protease inhibitors and centrifuged at 14,000 × *g* at 4 °C for 20 min. The supernatants were treated with 2% SDS and further heated at 100 °C for 15 min before incubation with the indicated primary antibodies at 4 °C for 4 h, and treatment with the Protein A-agarose at 4 °C overnight. Western blot was performed as previously described [36]. Primary antibodies including specific antibodies against Myc (16286-1-AP), RAC1 (24072-1-AP) and β-actin (66009-1-Ig) were purchased from Protein-tech (Philadelphia, PA, USA). Primary antibody against MG53 (ab238252) was purchased from Abcam (Cambridge, UK). Primary antibodies including specific antibodies against p-P38 (#4511), p-ERK (#4370) and p-JNK (#9255) were purchased from Cell Signaling Technology (Danvers, MA, USA).

### Immunofluorescence (IF) assay and in vitro protein translation assay

The immunofluorescence assay was performed as described before [48]. Antibodies were the same as those used for western blot. The in vitro protein translation assay was performed by TNT Quick Coupled Transcription and Translation System (Promega, Madison, WI, USA) as previously described [48].

### RAC1 GTPase activity assay

RAC1 GTPase activity assay was performed by using the RAC1 assay reagent (GST-PAK1-PBD on glutathione beads) according to the manufacturer’s instructions (Active RAC1 Activation Assay kit, Cell Signaling Technology, Danvers, MA, USA).

### In vitro ubiquitination assay

MG53 and RAC1 proteins were expressed in vitro by a TNT Quick Coupled Transcription and Translation System (Promega, Madison, WI, USA) according to the manufacturer’s protocol. In vitro ubiquitination assay was performed with a ubiquitination kit (Boston Biochem, MA, USA) following the manufacturer’s recommended protocol. The MG53 and RAC1 proteins were expressed in vitro separately and these two reaction mixtures were incubated in the Ubiquitin Conjugation Reaction Buffer (# B-70) containing ubiquitin (# Ue100H), UBE1 (# E1-305), UbcH5a (# E2-616), ATP and MgCl_2_ at 30 °C for 60 min.

### Clinical specimens

To evaluate the levels of MG53 and RAC1 in HCC patients, we detected the expression of MG53 and RAC1 in paired HCC tissues and corresponding distal non-cancerous liver tissues from 51 HCC patients in the Department of Hepatobiliary Surgery of the Provincial Hospital Affiliated to Shandong University. Written informed consents were obtained from all patients before participation. All protocols were in accordance with the Helsinki Declaration and were approved by Shandong University Research Ethics Committee.

### Proliferation, invasion and colony formation assay

The proliferation, invasion and colony formation assays of HCC cells were performed as previously described [36].

### Xenograft tumor model

4-week-old male BALB/c nude mice were used for the construction of xenografted tumor model in our investigation, and the xenograft tumor model in nude mice was constructed as described before [48]. When visible tumors appeared, the tumors at the left flanks were injected with 20 μg Myc-MG53 plasmid while tumors at the right flanks were injected with 20 μg Myc-vector plasmid before the mice were sacrificed on day 26 after the first injection. Another xenograft tumor model was constructed for investigation of the effective domain of MG53. When visible tumors appeared, the tumors were randomly divided into three groups including Myc-vector transfected group, Myc-MG53 transfected group and Myc-MG53-ΔRING transfected group. The tumors were injected with 20 μg of the plasmids every other day until the mice were sacrificed on day 28 for further analysis. All of the protocols carried out to mice were in accordance with the guidelines of the Institutional Animal Care and Use Committee, and approved by the Medical Ethics Committee of Shandong University.

### Statistical analysis

Data was presented as mean ± SD and analyzed by Prism GraphPad 5.0 (GraphPad Software, La Jolla, CA, USA) software. The compare of categorical variables was analyzed by Χ^2^-tests, and the continuous variables were analyzed by ANOVA and *T* test. Categorical variables were analyzed by Spearman rank correlation test, and continuous variables were analyzed by Person correlation test. All tests were two-tailed, and *P* < *0.05* was considered statistically significant.

## Supplementary information

Supplemental data

Conformation about author changes

## Data Availability

The datasets used and/or analyzed during this study are available from the corresponding author on reasonable request.

## References

[1] Kulik L, El-Serag HB (2019). Epidemiology and management of hepatocellular carcinoma. Gastroenterology.

[2] Llovet JM, Zucman-Rossi J, Pikarsky E, Sangro B, Schwartz M, Sherman M (2016). Hepatocellular carcinoma. Nat Rev Dis Prim.

[3] Llovet JM, Montal R, Sia D, Finn RS (2018). Molecular therapies and precision medicine for hepatocellular carcinoma. Nat Rev Clin Oncol.

[4] Nguyen LK, Kholodenko BN, von Kriegsheim A (2018). Rac1 and RhoA: Networks, loops and bistability. Small GTPases.

[5] Remorino A, De Beco S, Cayrac F, Di Federico F, Cornilleau G, Gautreau A (2017). Gradients of Rac1 nanoclusters support spatial patterns of Rac1 signaling. Cell Rep.

[6] Winge MC, Ohyama B, Dey CN, Boxer LM, Li W, Ehsani-Chimeh N (2016). RAC1 activation drives pathologic interactions between the epidermis and immune cells. J Clin Invest.

[7] Acevedo A, Gonzalez-Billault C (2018). Crosstalk between Rac1-mediated actin regulation and ROS production. Free Radic Biol Med.

[8] Yang J, Qiu Q, Qian X, Yi J, Jiao Y, Yu M (2019). Long noncoding RNA LCAT1 functions as a ceRNA to regulate RAC1 function by sponging miR-4715-5p in lung cancer. Mol Cancer.

[9] Zou T, Mao X, Yin J, Li X, Chen J, Zhu T (2017). Emerging roles of RAC1 in treating lung cancer patients. Clin Genet.

[10] Bayo J, Fiore EJ, Dominguez LM, Cantero MJ, Ciarlantini MS, Malvicini M (2021). Bioinformatic analysis of RHO family of GTPases identifies RAC1 pharmacological inhibition as a new therapeutic strategy for hepatocellular carcinoma. Gut.

[11] Takenaka N, Nihata Y, Ueda S, Satoh T (2017). In situ detection of the activation of Rac1 and RalA small GTPases in mouse adipocytes by immunofluorescent microscopy following in vivo and ex vivo insulin stimulation. Cell Signal.

[12] Akula MK, Ibrahim MX, Ivarsson EG, Khan OM, Kumar IT, Erlandsson M (2019). Protein prenylation restrains innate immunity by inhibiting Rac1 effector interactions. Nat Commun.

[13] Lorente M, Garcia-Casas A, Salvador N, Martinez-Lopez A, Gabicagogeascoa E, Velasco G, et al. Inhibiting SUMO1-mediated SUMOylation induces autophagy-mediated cancer cell death and reduces tumour cell invasion via RAC1. 2019;132:jcs234120, 1–12. 10.1242/jcs.234120 PMC6826015 31578236

[14] Oberoi-Khanuja TK, Rajalingam K (2014). Ubiquitination of Rac1 by inhibitors of apoptosis (IAPs). Methods Mol Biol.

[15] Oberoi TK, Dogan T, Hocking JC, Scholz RP, Mooz J, Anderson CL (2012). IAPs regulate the plasticity of cell migration by directly targeting Rac1 for degradation. EMBO J.

[16] Torrino S, Visvikis O, Doye A, Boyer L, Stefani C, Munro P (2011). The E3 ubiquitin-ligase HACE1 catalyzes the ubiquitylation of active Rac1. Dev Cell.

[17] Li T, Qin JJ, Yang X, Ji YX, Guo F, Cheng WL (2017). The ubiquitin E3 ligase TRAF6 exacerbates ischemic stroke by ubiquitinating and activating Rac1. J Neurosci.

[18] Frances D, Sharma N, Pofahl R, Maneck M, Behrendt K, Reuter K (2015). A role for Rac1 activity in malignant progression of sebaceous skin tumors. Oncogene.

[19] McBeath R, Edwards RW, O’Hara BJ, Maltenfort MG, Parks SM, Steplewski A (2019). Tendinosis develops from age- and oxygen tension-dependent modulation of Rac1 activity. Aging Cell.

[20] Marston DJ, Anderson KL, Swift MF, Rougie M, Page C, Hahn KM (2019). High Rac1 activity is functionally translated into cytosolic structures with unique nanoscale cytoskeletal architecture. Proc Natl Acad Sci USA.

[21] Cai C, Masumiya H, Weisleder N, Matsuda N, Nishi M, Hwang M (2009). MG53 nucleates assembly of cell membrane repair machinery. Nat Cell Biol.

[22] Liu W, Wang G, Zhang C, Ding W, Cheng W, Luo Y (2019). MG53, A novel regulator of KChIP2 and Ito,f, plays a critical role in electrophysiological remodeling in cardiac hypertrophy. Circulation.

[23] Bian Z, Wang Q, Zhou X, Tan T, Park KH, Kramer HF (2019). Sustained elevation of MG53 in the bloodstream increases tissue regenerative capacity without compromising metabolic function. Nat Commun.

[24] Wu HK, Zhang Y, Cao CM, Hu X, Fang M, Yao Y (2019). Glucose-sensitive myokine/cardiokine MG53 regulates systemic insulin response and metabolic homeostasis. Circulation.

[25] Sermersheim M, Kenney AD, Lin PH, McMichael TM, Cai C, Gumpper K (2020). MG53 suppresses interferon-beta and inflammation via regulation of ryanodine receptor-mediated intracellular calcium signaling. Nat Commun.

[26] Yi JS, Park JS, Ham YM, Nguyen N, Lee NR, Hong J (2013). MG53-induced IRS-1 ubiquitination negatively regulates skeletal myogenesis and insulin signalling. Nat Commun.

[27] Jaworska AM, Wlodarczyk NA, Mackiewicz A, Czerwinska P (2020). The role of TRIM family proteins in the regulation of cancer stem cell self-renewal. Stem Cells.

[28] Hatakeyama S (2017). TRIM family proteins: roles in autophagy, immunity, and carcinogenesis. Trends Biochem Sci.

[29] Song R, Peng W, Zhang Y, Lv F, Wu HK, Guo J (2013). Central role of E3 ubiquitin ligase MG53 in insulin resistance and metabolic disorders. Nature..

[30] Lionarons DA, Hancock DC, Rana S, East P, Moore C, Murillo MM (2019). RAC1(P29S) induces a mesenchymal phenotypic switch via serum response factor to promote melanoma development and therapy resistance. Cancer Cell.

[31] Jiang ZB, Ma BQ, Liu SG, Li J, Yang GM, Hou YB (2019). miR-365 regulates liver cancer stem cells via RAC1 pathway. Mol Carcinog.

[32] Wei L, Lee D, Law CT, Zhang MS, Shen J, Chin DW (2019). Genome-wide CRISPR/Cas9 library screening identified PHGDH as a critical driver for Sorafenib resistance in HCC. Nat Commun.

[33] Zhu YJ, Zheng B, Wang HY, Chen L (2017). New knowledge of the mechanisms of sorafenib resistance in liver cancer. Acta Pharm Sin.

[34] Li Q, Ren B, Gui Q, Zhao J, Wu M, Shen M (2020). Blocking MAPK/ERK pathway sensitizes hepatocellular carcinoma cells to temozolomide via downregulating MGMT expression. Ann Transl Med.

[35] Dietrich P, Koch A, Fritz V, Hartmann A, Bosserhoff AK, Hellerbrand C (2018). Wild type Kirsten rat sarcoma is a novel microRNA-622-regulated therapeutic target for hepatocellular carcinoma and contributes to sorafenib resistance. Gut.

[36] Zhang Y, Li T, Guo P, Kang J, Wei Q, Jia X (2014). MiR-424-5p reversed epithelial-mesenchymal transition of anchorage-independent HCC cells by directly targeting ICAT and suppressed HCC progression. Sci Rep.

[37] Bao Z, Zhang L, Li L, Yan J, Pang Q, Sun Z (2020). Nepsilon-carboxymethyl-lysine negatively regulates foam cell migration via the Vav1/Rac1 pathway. J Immunol Res.

[38] Navarro-Lerida I, Sanchez-Perales S, Calvo M, Rentero C, Zheng Y, Enrich C (2012). A palmitoylation switch mechanism regulates Rac1 function and membrane organization. EMBO J.

[39] Chen Z, Yin X, Li K, Chen S, Li H, Li Y (2018). Serum levels of TRIM72 are lower among patients with colon cancer: identification of a potential diagnostic marker. Tohoku J Exp Med.

[40] Yin W, Liu Y, Bian Z (2019). MG53 inhibits the progression of tongue cancer cells through regulating PI3K-AKT signaling pathway: evidence from 3D cell culture and animal model. Small.

[41] De P, Rozeboom BJ, Aske JC, Dey N. Active RAC1 promotes tumorigenic phenotypes and therapy resistance in solid tumors. Cancers (Basel). 2020;12:1541, 1–16. 10.3390/cancers12061541 PMC7352592 32545340

[42] Kim EK, Choi EJ (2010). Pathological roles of MAPK signaling pathways in human diseases. Biochim Biophys Acta.

[43] Tu Z, Wang Q, Cui T, Wang J, Ran H, Bao H (2016). Uterine RAC1 via Pak1-ERM signaling directs normal luminal epithelial integrity conducive to on-time embryo implantation in mice. Cell Death Differ.

[44] Zhou H, Cai L, Zhang X, Li A, Miao Y, Li Q (2018). ARHGEF39 promotes tumor progression via activation of Rac1/P38 MAPK/ATF2 signaling and predicts poor prognosis in non-small cell lung cancer patients. Lab Invest.

[45] Jiang K, Lu Q, Li Q, Ji Y, Chen W, Xue X (2017). Astragaloside IV inhibits breast cancer cell invasion by suppressing Vav3 mediated Rac1/MAPK signaling. Int Immunopharmacol.

[46] Ma X, Qiu Y, Sun Y, Zhu L, Zhao Y, Li T (2020). NOD2 inhibits tumorigenesis and increases chemosensitivity of hepatocellular carcinoma by targeting AMPK pathway. Cell Death Dis.

[47] Wei Q, Guo P, Mu K, Zhang Y, Zhao W, Huai W (2015). Estrogen suppresses hepatocellular carcinoma cells through ERbeta-mediated upregulation of the NLRP3 inflammasome. Lab Invest.

[48] Guo P, Ma X, Zhao W, Huai W, Li T, Qiu Y (2018). TRIM31 is upregulated in hepatocellular carcinoma and promotes disease progression by inducing ubiquitination of TSC1-TSC2 complex. Oncogene.

