# Supplementary material for: The E3 ubiquitin ligase MG53 inhibits hepatocellular carcinoma by targeting RAC1 signaling
Source: Oncogenesis. 2022 Jul 20;11(1):40. doi: 10.1038/s41389-022-00414-6 (PMC9300626; doi:10.1038/s41389-022-00414-6)
Supplement: Supplementary file 1 — Supplemental data [file 41389_2022_414_MOESM1_ESM.docx]

**Supplementary Information**

[**The E3 ubiquitin ligase MG53**](https://www.ncbi.nlm.nih.gov/pubmed/25701873) **inhibits hepatocellular carcinoma by targeting RAC1 signaling**

**Running Title: MG53 inhibits hepatocellular carcinoma by targeting RAC1 signaling**

Xiaomin Ma^1,2^, Xiaoxiao Ma^1^, Lihui Zhu^1^, Yunxue Zhao^3^, Mengmeng Chen ^4^, Tao Li^5^, Yueke Lin^1^, Dapeng Ma^1^, Caiyu Sun^1^, and Lihui Han^1^*

1. Shandong Provincial Key Laboratory of Infection & Immunology, Shandong Provincial Clinical Research Center for Immune Diseases and Gout, Department of Immunology, School of Basic Medical Sciences, Cheeloo college of Medicine, Shandong University, Jinan 250012, China.
2. Department of General Surgery, The First Affiliated Hospital of Shandong First Medical University & Shandong Provincial Qianfoshan Hospital, Jinan 250014, Shandong Province, China.
3. Department of Pharmacology, Shandong University School of Basic Medical Sciences, Jinan 250012, China.
4. Qingdao Restore Biotechnology Co., Ltd., Qingdao, Shandong 266111, P.R. China
5. Department of Infectious diseases, Shandong Provincial Hospital Affiliated to Shandong First Medical University, Jinan 250021, China.

**Contact information：**Lihui Han, M.D., Ph.D., Department of Immunology, Shandong University School of Basic Medical Sciences, 44 Wenhua Xi Road, Jinan 250012, China. Phone: 86-531-88382038. Fax: 86-531-8832038. E-Mail: [hanlihui@sdu.edu.cn](mailto:hanlihui@sdu.edu.cn)

**Supplemental Table 1.**

**Clinicopathological characteristics of the investigated HCC patients**

| **Characteristics** | **for qRT-PCR and western blot; n=51** |
| --- | --- |
| **Gender** |  |
| Male | 39 (76.5%) |
| Female | 12 (23.5%) |
| **Age** |  |
| <54 | 20 (39.2%) |
| ≥54 | 31 (60.8%) |
| **Liver cirrhosis history** |  |
| Yes | 17 (33.3%) |
| No | 34 (66.7%) |
| **TNM stage** |  |
| I | 11 (21.5%) |
| II | 23 (45.1%) |
| III | 16 (31.4%) |
| IV | 1 (2%) |
| **Regional lymph nodes** |  |
| N0 | 24 (47.1%) |
| N1 | 27 (52.9%) |
| **BCLC stages** |  |
| 0 | 20 (39.2%) |
| A | 23 (45.1%) |
| B | 7 (13.7%) |
| C | 1 (2%) |
| D | 0 |
| **Distant metastasis** |  |
| No | 20(39.2%) |
| Yes | 31(60.8%) |
|  |  |

**Supplemental Figures**

**Figure S1**

**
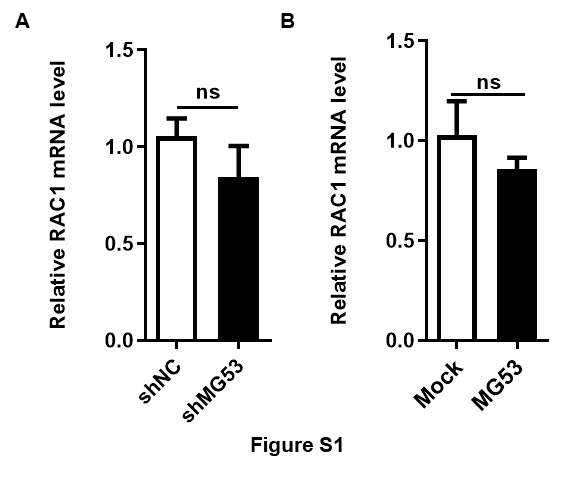
**

**Figure S2**

**
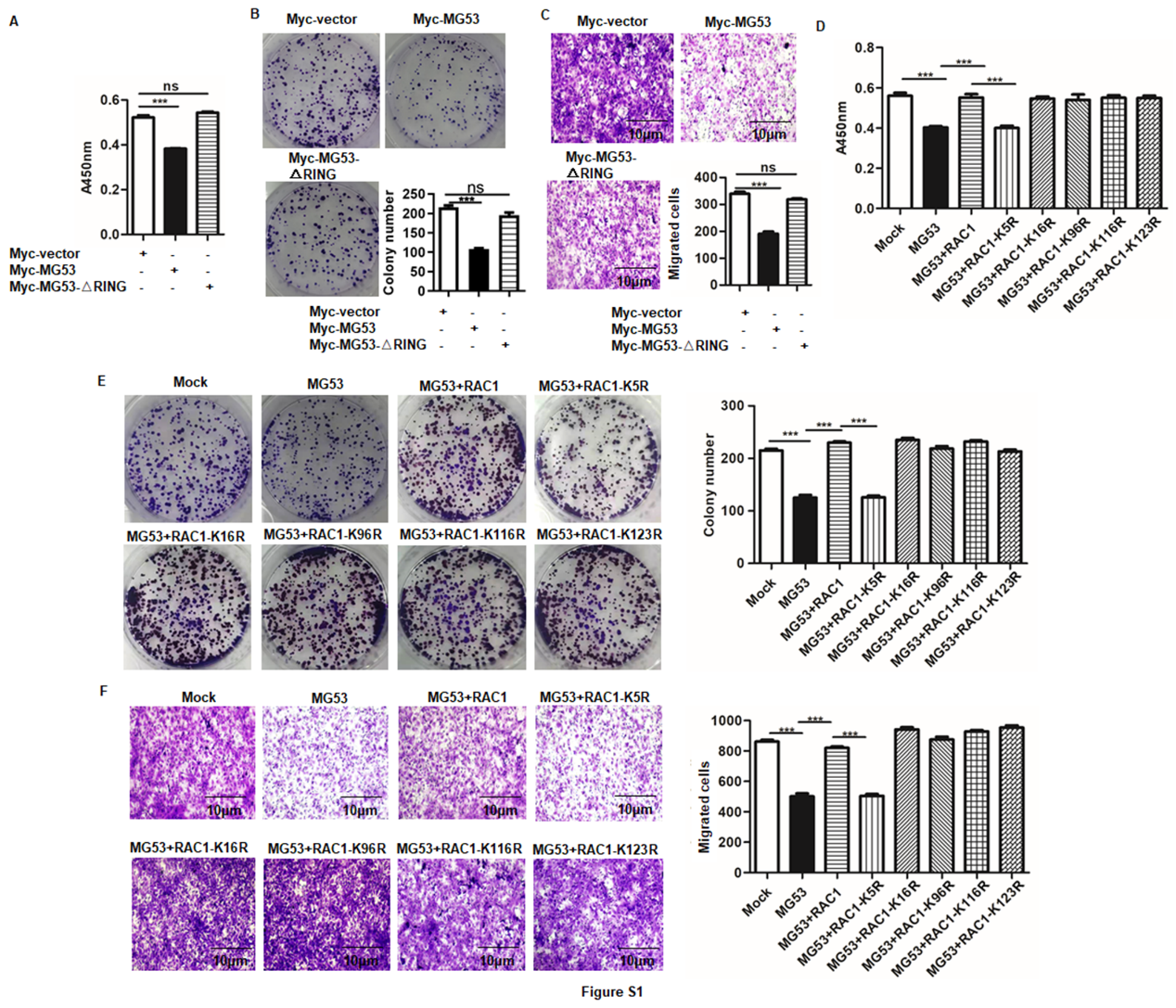
**

**Figure S3**


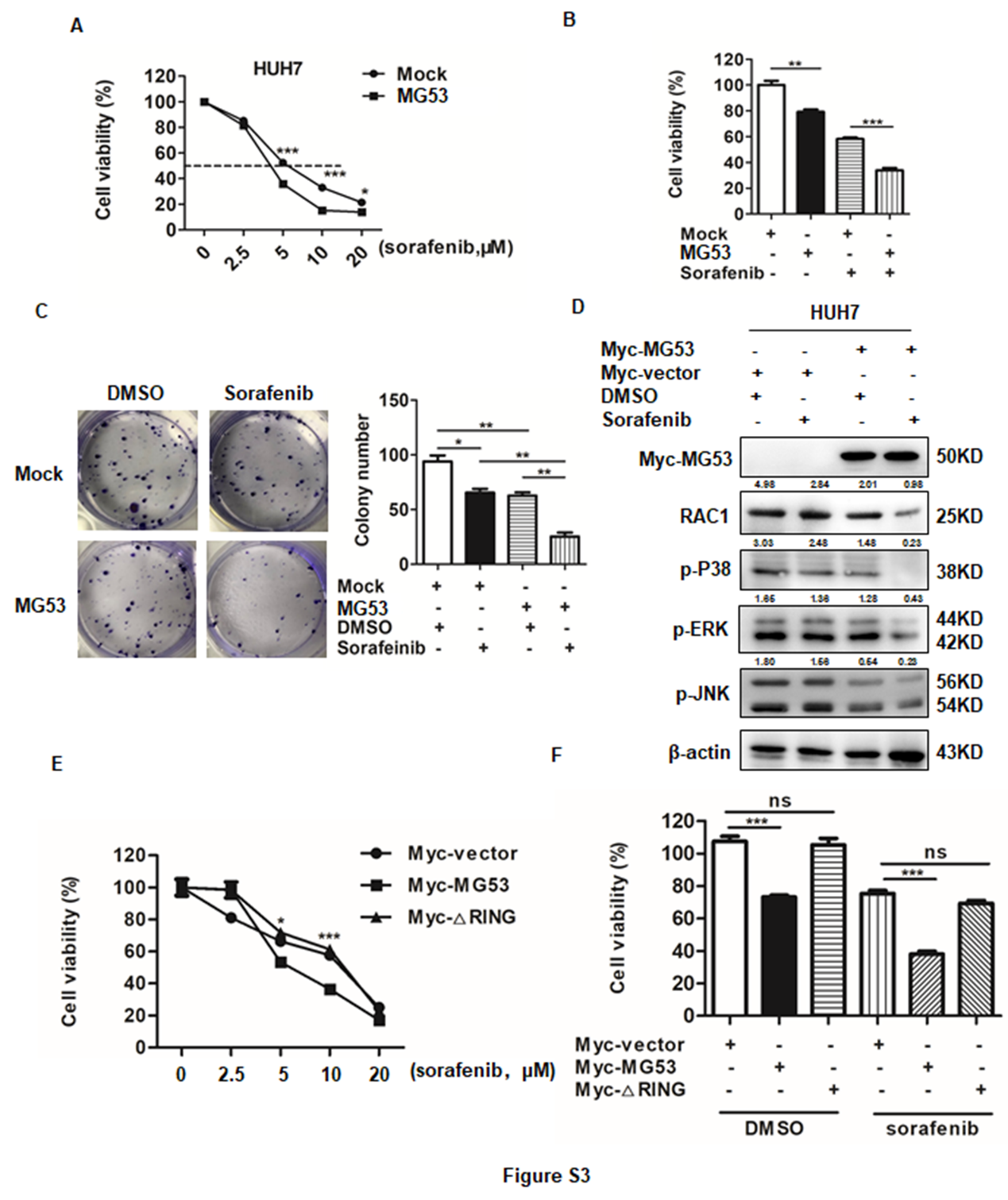


**Supplemental Figure legends**

**Figure S1. MG53 had no effect on RAC1 mRNA expression. (A)** HUH7 cells were transfected with shMG53 plasmid, and shNC plasmid transfected cells acted as the control. qRT-PCR was performed to detect the mRNA level of RAC1 of these transfected cells 48h after the transfection. **(B)** HUH7 cells were transfected with Myc-MG53 plasmid, and Myc-vector plasmid transfected cells acted as the mock control. qRT-PCR was performed to detect the mRNA level of RAC1 in these transfected cells 48h after the transfection.

**Figure S2. MG53 inhibited the malignant behaviors of HCC via its RING domain and abolished RAC1 function at Lys5 residue. (A-C)** HUH7 cells were transfected with Myc-MG53 or its RING domain delated mutant plasmid, and the vector plasmid transfected cells acted as the mock control. CCK-8 assay was performed to determine the proliferation of these transfected cells (A). Colony formation assay was performed to detect the proliferation of these transfected HCC cells (B). Transwell assay was performed to detect the invasion of these transfected HCC cells (C). **(D-F)** HUH7 cells were co-transfected with Myc-MG53 and Flag-RAC1 or RAC1 mutants, and cells co-transfected with Myc-vector and Flag-RAC1 acted as the mock control. CCK-8 assay was performed to detect the proliferation of these transfected cells 48h after the transfection (D). Colony formation assay was performed to detect the proliferation of these transfected HCC cells (E). Transwell assay was performed to detect the invasion of these transfected HCC cells (F). **P*<0.05, ***P*<0.01 and ****P*<0.001 for statistical analysis of the indicated groups.

**Figure S3.** **MG53 significantly enhanced the chemosensitivity of HCC cells to sorafenib. (A)** HUH7 cells were plated in 96-well plate at the density of 8 X10^3^ cells/well. After being cultured overnight, the cells were transfected with myc-MG53 or mock control. The transfected cells were further treated with different dosages (0μM, 2.5μM, 5μM, 10μM and 20μM) of sorafenib. 48h after the treatment, CCK-8 assay was performed to detect the cell viabilities of the treated HUH7 cells. **(B)** HUH7 cells were plated in 96-well plate before being transfected with Myc-MG53 or mock control. HCC cells were treated with sorafenib (5μM), and the cells treated with the same volume of DMSO acted as a vehicle control. Cell viabilities of the treated HUH7 cells were detected by CCK-8 assay 48h after the treatment. **(C)** HUH7 cells were plated in the 6-well plate before being transfected with Myc-MG53 or mock control. 6h after the transfection, cells were treated with sorafenib (5μM), and the cells with the treatment of the same volume of DMSO acted as a vehicle control. 24h after the treatment, cells were collected for the colony formation assay. Sorafenib was added to the cells every 72h, and colony number was counted 8 days after the transfection. **(D)** HUH7 cells were transfected with Myc-MG53 or mock control, followed by further treatment with sorafenib (5μM) for 48h. Western blot assay was performed to detect the levels of RAC1, p-P38, p-ERK and p-JNK. **(E)** HUH7 cells were plated in the 96-well plate at the density of 8 X10^3^ cells/well. After being cultured overnight, the cells were transfected with Myc-MG53 or the RING domain deleted MG53 mutant, and cells transfected with Myc-vector acted as the mock control. The transfected cells were further treated with different dosages (0μM, 2.5μM, 5μM, 10μM and 20μM) of sorafenib. 48h after the treatment, CCK-8 assay was performed to detect the cell viabilities of the treated HUH7 cells. **(F)** HUH7 cells were plated in the 96-well plate before being transfected with Myc-MG53 or the RING domain deleted MG53 mutant, and cells transfected with Myc-vector acted as the mock control. HCC cells were treated with sorafenib (5μM), and cells treated with the same volume of DMSO acted as a vehicle control. Cell viabilities of the treated HUH7 cells were detected by CCK-8 assay 48h after the treatment. **P*<0.05, ***P*<0.01 and ****P*<0.001 for statistical analysis of the indicated groups.
