# Supplementary material for: The E3 ubiquitin ligase MG53 inhibits hepatocellular carcinoma by targeting RAC1 signaling
Source: Oncogenesis. 2022 Jul 20;11(1):40. doi: 10.1038/s41389-022-00414-6 (PMC9300626; doi:10.1038/s41389-022-00414-6)

**Agreement response from Dr. Xiaomin Ma, the First Author**


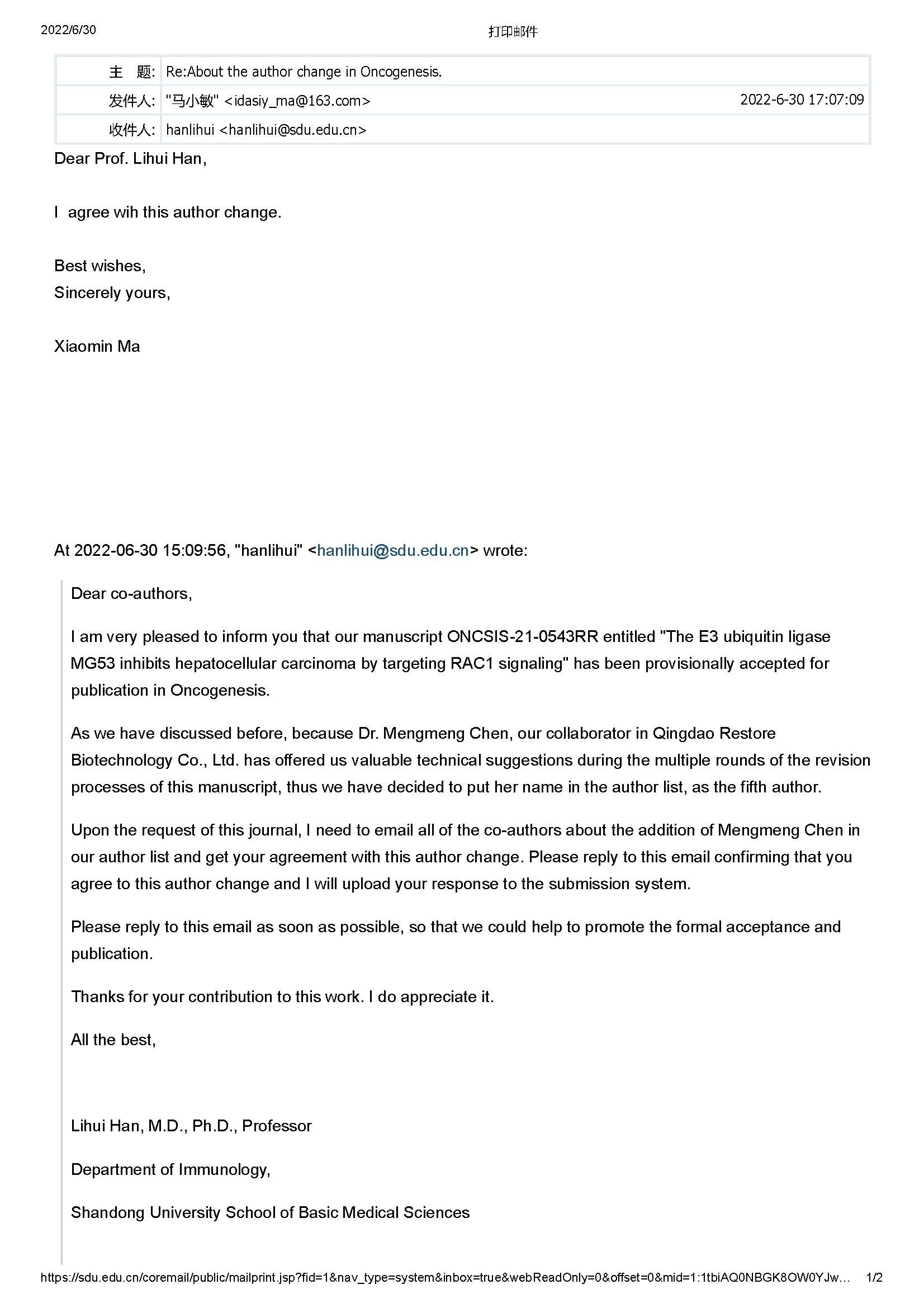


**Agreement response from Dr. Xiaoxiao Ma, the Second Author**


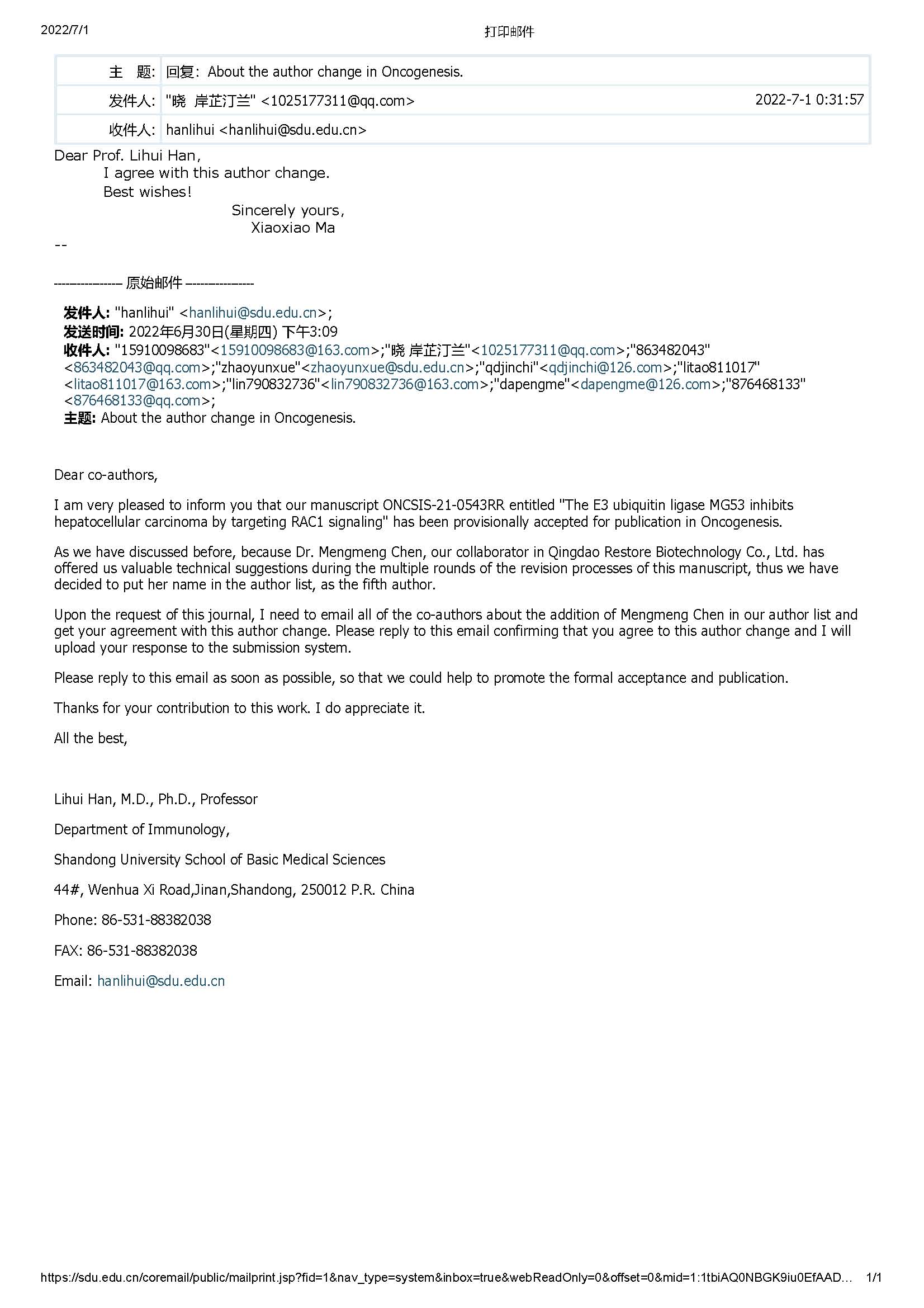


**Agreement response from Dr. Lihui Zhu, the Third Author**


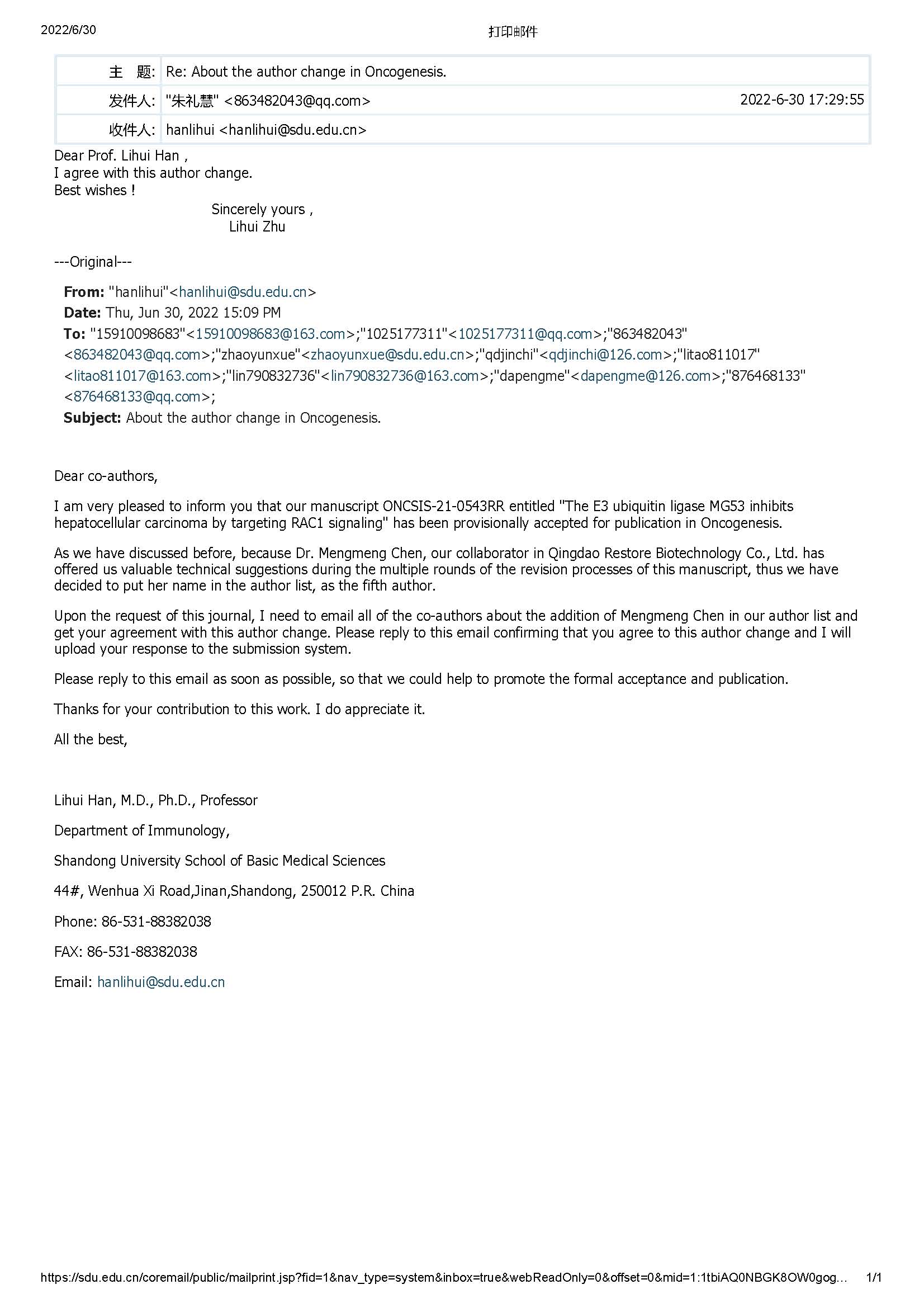


**Agreement response from Dr. Yunxue Zhao, the Forth Author**


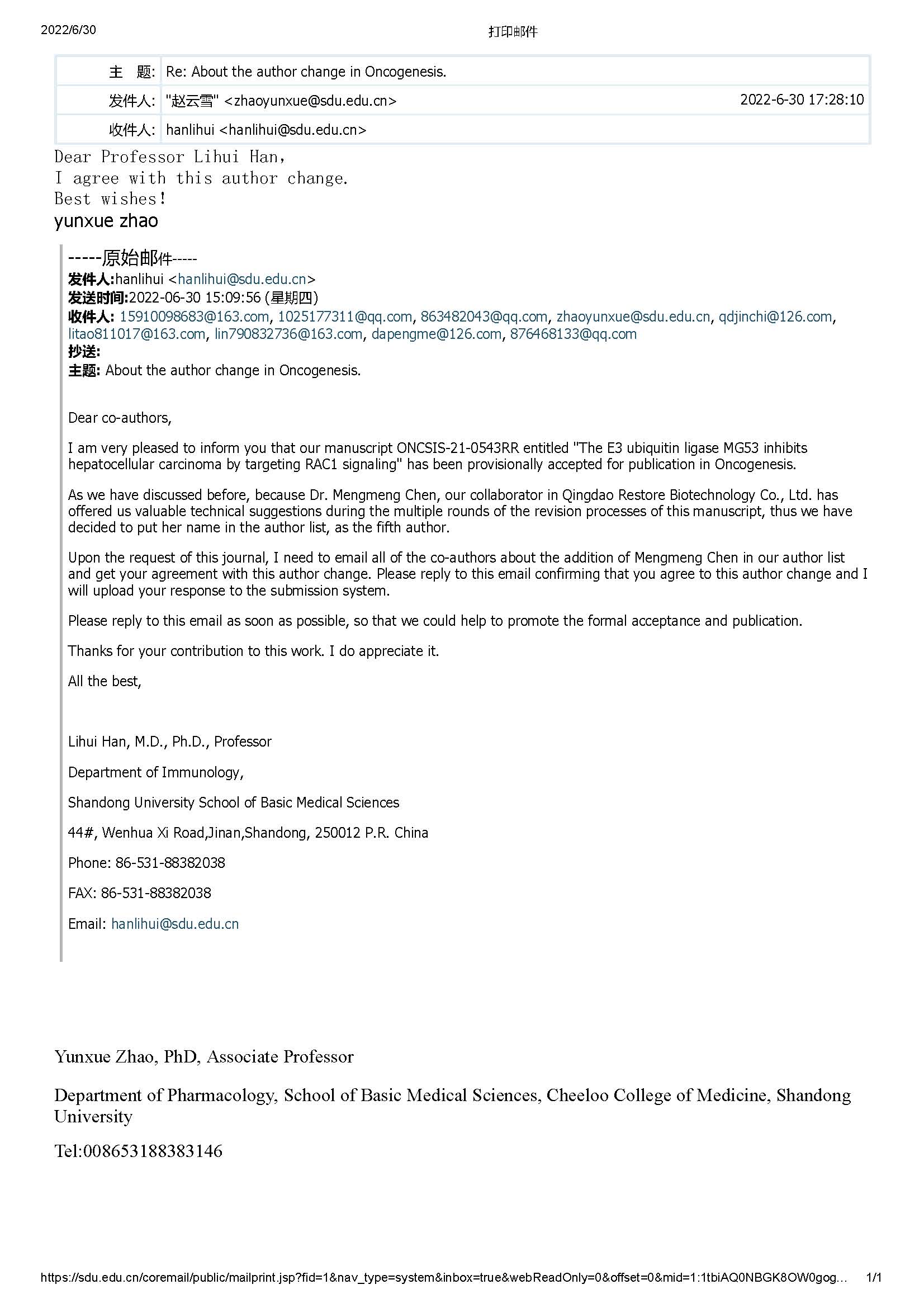


**Agreement response from Dr. Mengmeng Chen, the Fifth Author**

**
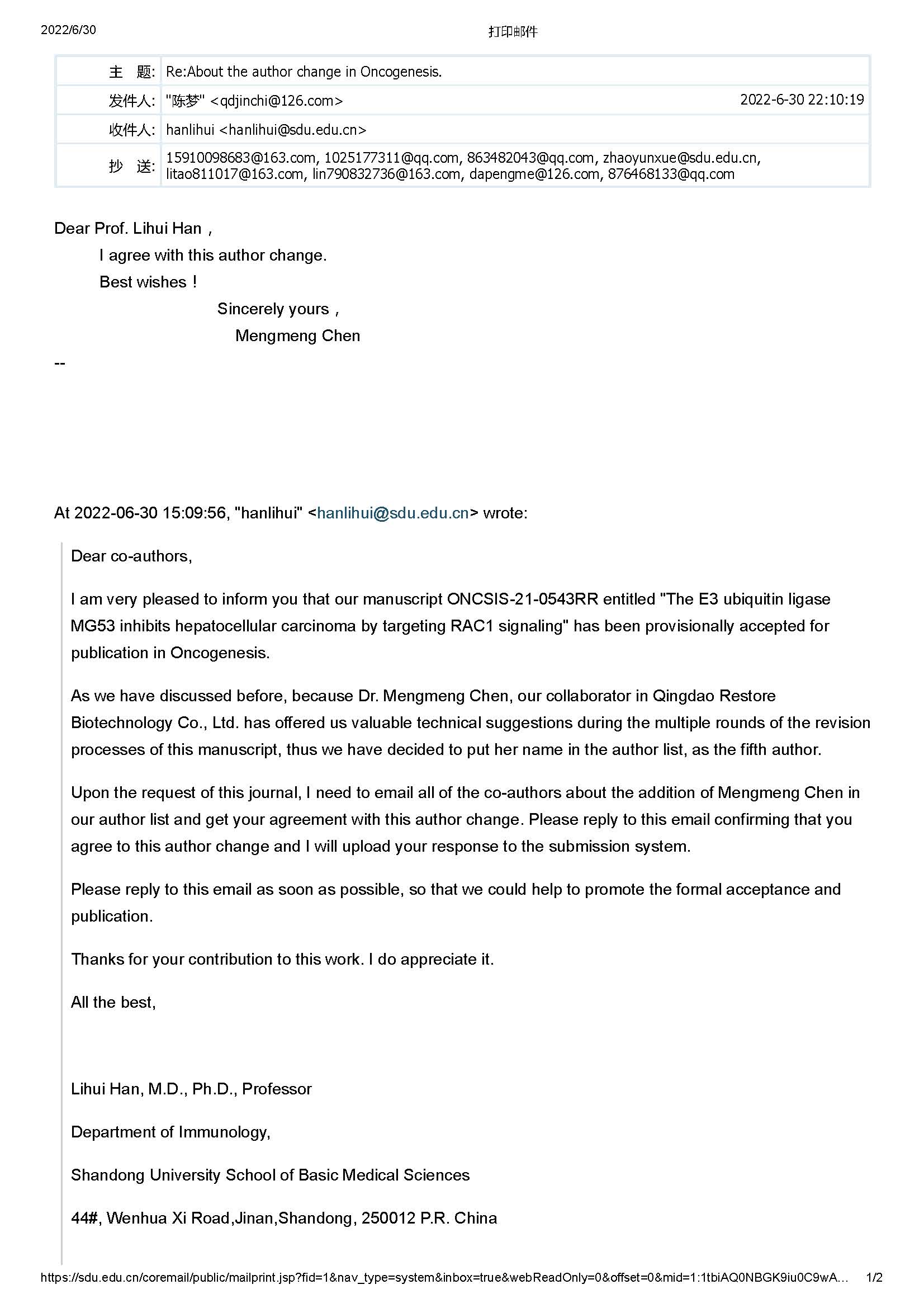
**

**Agreement response from Dr. Tao Li, the Sixth Author**

**
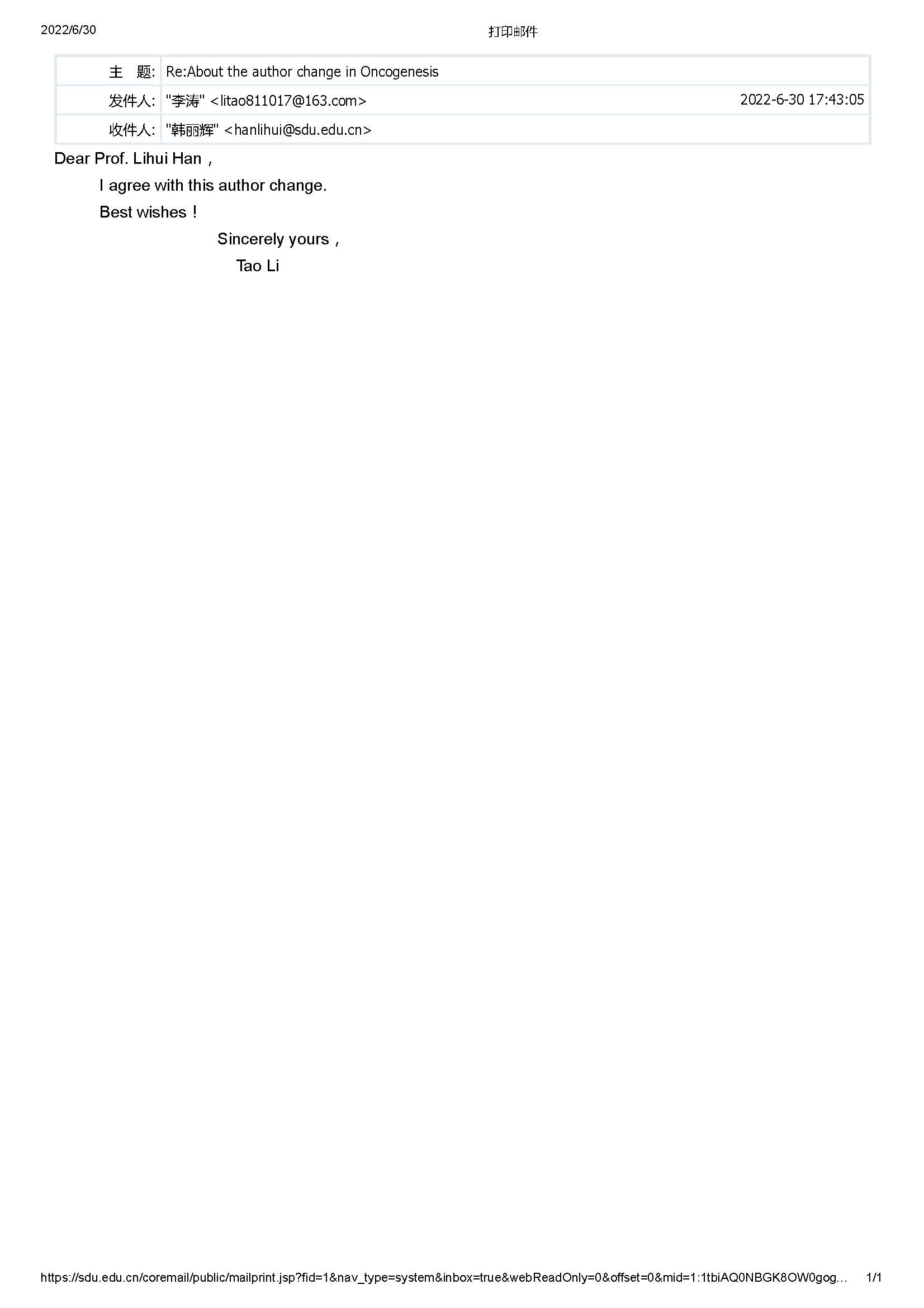
**

**Agreement response from Dr. Yueke Lin, the Seven Author**

**
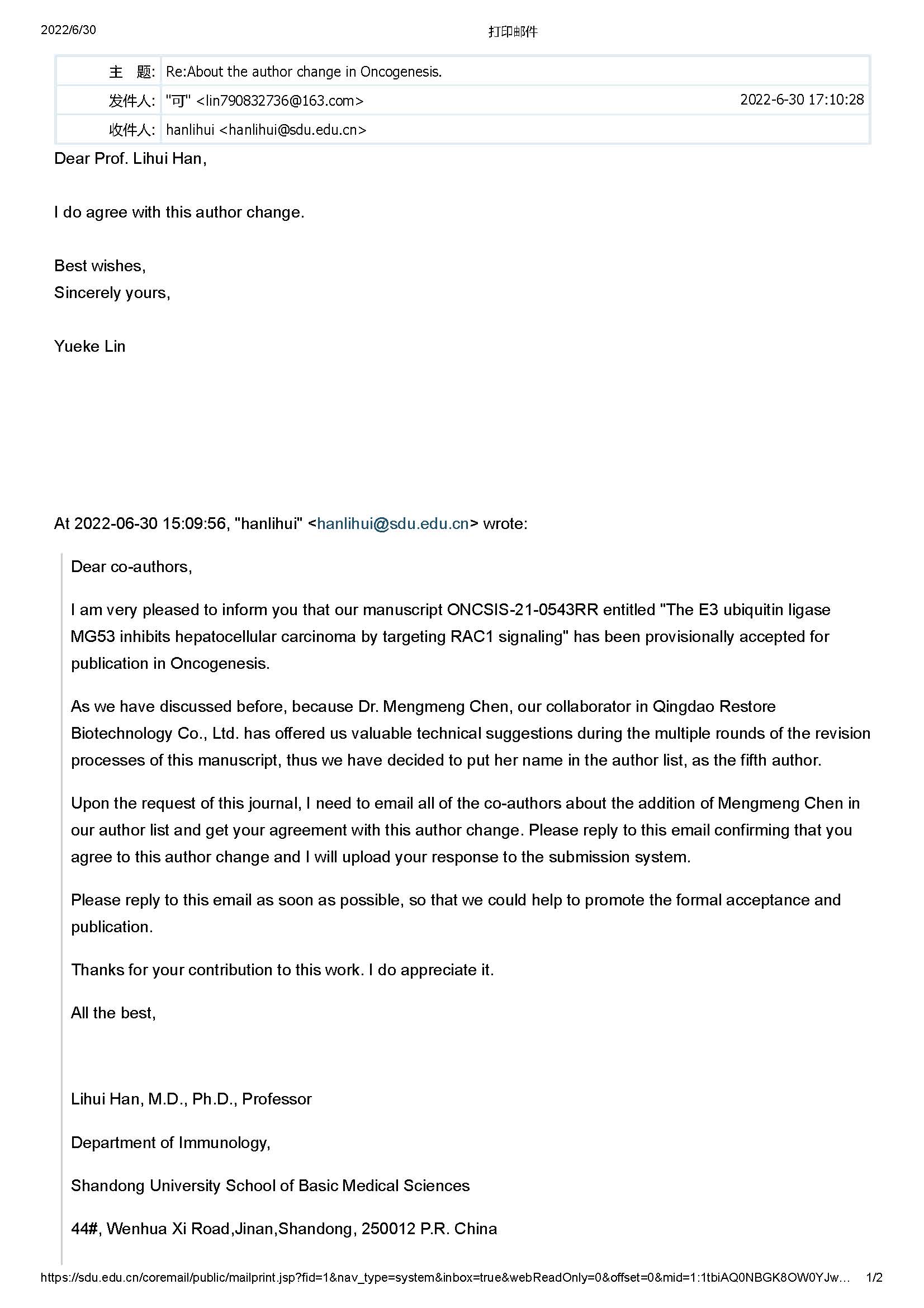
**

**Agreement response from Dr.Dapeng Ma, the Eighth Author**

**
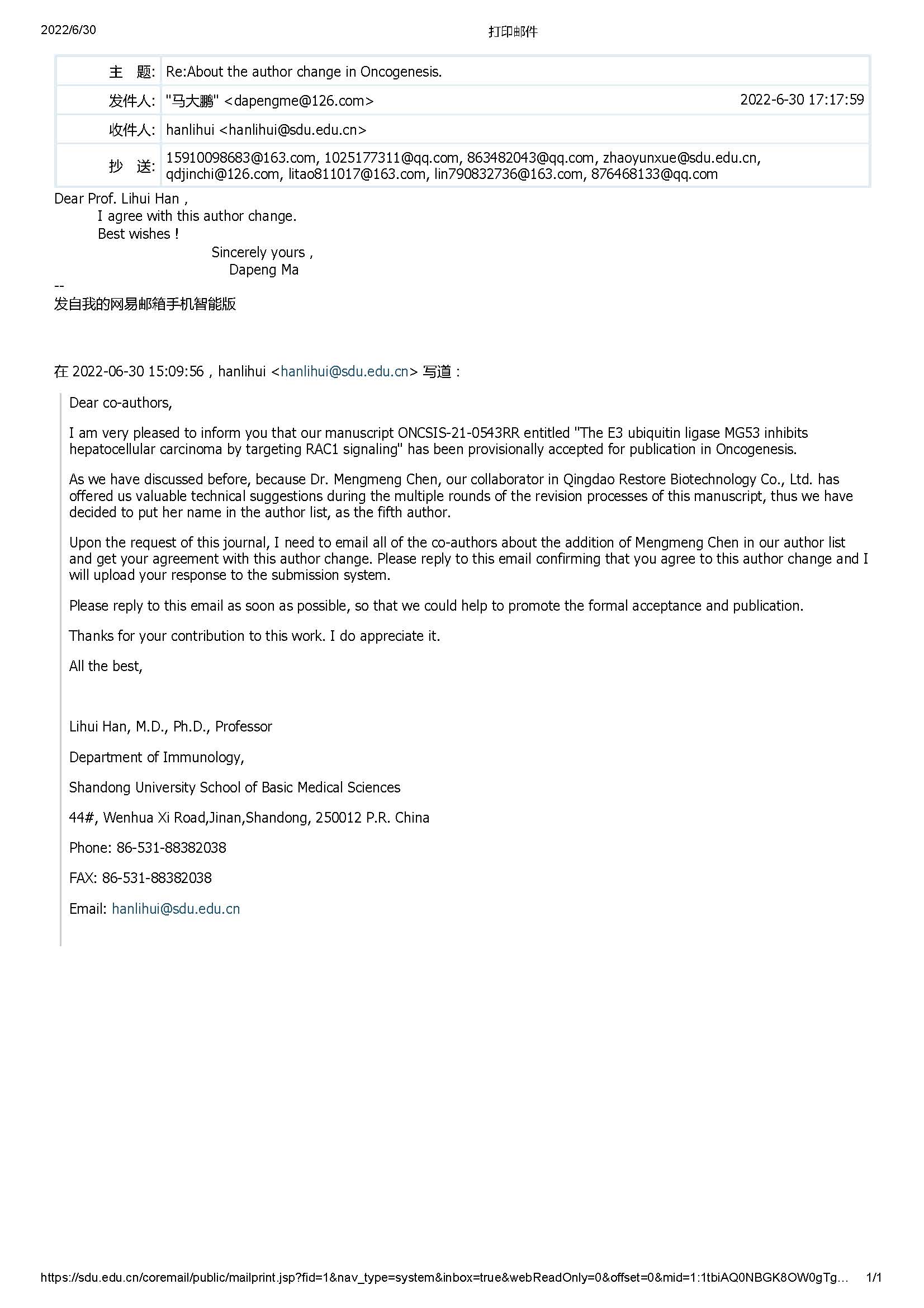
**

**Agreement response from Dr. Caiyu Sun, the Ninth Author**


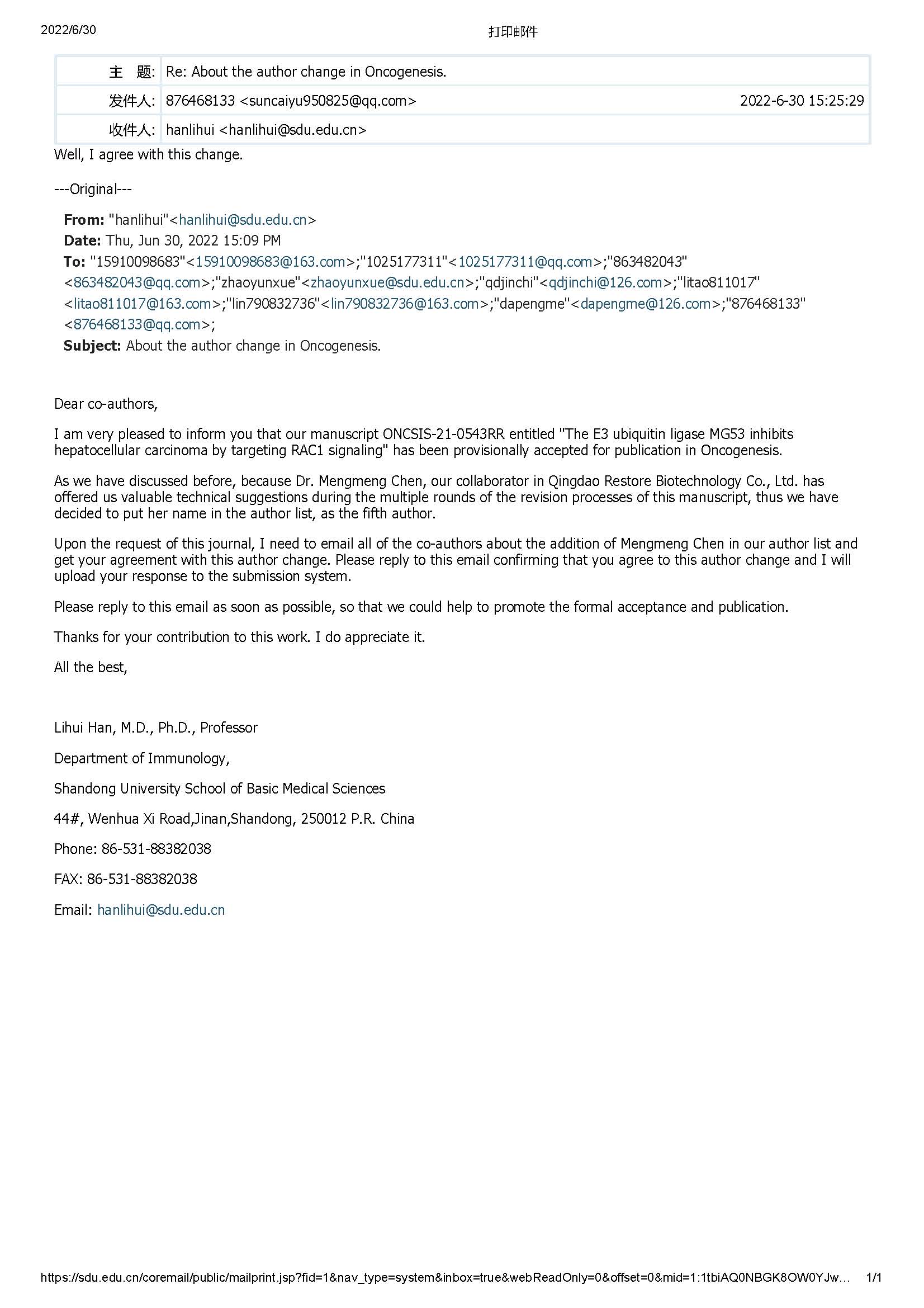

Supplement: Supplementary file 2 — Conformation about author changes [file 41389_2022_414_MOESM2_ESM.docx]
